# Improving the Activity of Antimicrobial Peptides Against Aquatic Pathogen Bacteria by Amino Acid Substitutions and Changing the Ratio of Hydrophobic Residues

**DOI:** 10.3389/fmicb.2021.773076

**Published:** 2021-10-18

**Authors:** Rong Tan, Meiru Wang, Huiqin Xu, Lu Qin, Jun Wang, Pengfei Cui, Shaoguo Ru

**Affiliations:** College of Marine Life Science, Ocean University of China, Qingdao, China

**Keywords:** antimicrobial peptides (AMP), antibiotics, aquatic pathogen bacteria, structure-based design, amino acid substitutions

## Abstract

With the increasing number of drug-resistant bacteria, there is an urgent need for new antimicrobial agents, and antimicrobial peptides (AMPs), which exist in the human non-specific immune system, are one of the most promising candidates. It is an effective optimization strategy to modify antimicrobial peptides (AMPs) according to the distribution of amino acids and hydrophobic characteristics. The addition of bacterial pheromones to the N short peptide can increase the ability to recognize bacteria. In this study, we designed and synthesized AMP1–6 by amino acid substitution of mBjAMP1. Additionally, P-6, S-6, and L-6 were designed and synthesized by adding bacterial pheromones based on 1–6. Functional tests showed that the four AMPs had the ability to kill Gram-negative *Vibrio anguillarum*, *Pseudomonas mendocina*, and *Vibrio parahaemolyticus*, and Gram-positive *Micrococcus luteus* and *Listeria monocytogenes*. Additionally, all four AMPs induced permeabilization and depolarization of bacterial cell membranes and increased intracellular reactive oxygen species (ROS) levels. Importantly, they had little or no mammalian cytotoxicity. At the same time, 1–6 and L-6 protected the stability of intestinal flora in *Sebastes schlegelii* and increased the relative abundance of *Lactobacillaceae*. In summary, our results indicate that the designed AMPs have broad application prospects as a new type of polypeptide antimicrobial agent.

## Introduction

Multidrug-resistant bacteria (MRB) and the spread of bacterial resistance to all routinely used antibiotics caused by the use and misuse of antibiotics are emerging as major global health concerns and are regarded as one of the biggest threats to humankind in the future. Currently, infections caused by antibiotic-resistant bacteria are estimated to cause more than 700,000 deaths annually, and this number is increasing. The use of antibiotics leads to increasing types and abundance of drug-resistant *Vibrio parahaemolyticus*, one of the most harmful aquatic pathogens that can induce food poisoning. *Pseudomonas mendocina* can cause endocarditis or sepsis. Many species of this genus are resistant to antibiotics and have a variety of virulence factors (Moradali et al., 2017). *Listeria monocytogenes* can also lead to sepsis, meningitis and so on. *Vibrio anguillarum* is the main pathogen of epizootic disease in marine fish. This bacterium affects a large number of wild and farmed fish, causing huge economic losses to the aquaculture industry (Torres et al., 2021). *Micrococcus luteus* can cause internal organs and body surface hemorrhage of fish, which is a threat to human and animal health. With the ever-growing global concerns about MRB, there is an urgent demand for the exploration of new antibiotics, such as antimicrobial peptides (AMPs), biometallic organic molecules (Tacke, 2015), natural plants and extracts (Pan et al., 2003), nanoparticles (Qing et al., 2018), and enzyme preparation. In the last three decades, AMPs have gained increasing attention as promising candidates to overcome the challenges of antibiotic resistance (Bahar and Ren, 2013).

Antimicrobial peptides generally exhibit a wide range of antimicrobial activities as antimicrobial, antifungal, and antiparasitic agents and are usually highly membrane-selective (Splith and Neundorf, 2011; Semreen et al., 2018). Natural AMPs generally contain less than 60 amino acids, a considerable part of hydrophobic residues (Arias et al., 2018), and are positively charged with a charge range of +2 to +9.

Antimicrobial peptides are usually cationic and amphipathic molecules that interact with microbial membranes or kill microbes by direct disruption of cellular components, including the microbial membrane, DNA, and proteins (Brogden, 2005; Wang et al., 2015). A variety of AMPs have an α-helical structure, composed of hydrophobic and cationic amino acid residues arranged on one side of the helix (Avitabile et al., 2013; Baeriswyl et al., 2019). These AMPs usually interact with negatively charged bacterial membranes through cationic amino acids, resulting in their insertion into the membranes and inducing lysis of the bacterial membranes (Karmakar et al., 2019). Therefore, the possibility of generating MRB against AMPs has been recognized to be low, in sharp contrast to conventional antibiotics, owing to their various mechanisms. The mechanism of action involves cell membrane rupture without any receptor-mediated pathway and without any target, thus impeding the development of AMP-resistant microorganisms (Yount et al., 2006; Enoki et al., 2018). Therefore, AMPs may be a promising candidate for MRB therapy, which is of great significance in dealing with a range of problems caused by antibiotic overuse (Ageitos et al., 2017).

However, to date, no AMP has reached the antibiotic market, although many AMPs are currently in clinical trials (Greber and Dawgul, 2017). The biggest challenges faced in the development of AMPs into drugs are high production costs (especially for large and disulfide-rich peptides), lack of proteolytic stability, and unpredictable toxicology profile when administered systemically (Fjell et al., 2011; Ramesh et al., 2016). These characteristics limit their application. To solve this problem, it is necessary to design and modify natural AMPs. Mourtada et al. (2019) designed Mag (i +4), with low hemolytic activity, high stability, and strong antibacterial activity, by continuously optimizing the natural AMP magainin II (MAG2) by replacing amino acids, increasing hydrophobic proportions, and analyzing hydrophobic distribution and strength.

Bacterial pheromones are specific signaling molecules in some bacteria that mediate intercellular communication (Yajima, 2014). These pheromones can cross the cell wall and bind to homologous membrane receptors with high affinity (Lyon and Novick, 2004). The significant affinity between pheromones and membrane receptors makes them ideal candidates for promoting AMP aggregation and enhancing antimicrobial activity. In fact, recent studies have shown that potent, short (<15 amino acids), linear AMPs can be successfully produced (Mikut et al., 2016; Ramesh et al., 2016). Xu et al. (2020) successfully constructed *Enterococcus faecalis*-targeted AMPs by adding *E. faecalis* pheromone. Certain characteristics have also been shown to play a critical role in the activity of these peptides, such as the balance among the positive charge, hydrophobicity, and content of lipophilic bulky residues, such as Trp (Mikut et al., 2016).

In this study, we initially identified the physical and chemical properties of AMP1–6 and AMP1–6 derivatives, and then investigated their antimicrobial, hemolytic, and cell-membrane disruption activities and the effects on intestinal microbial diversity in fish. The aim of this study was to design and modify AMP molecules through bioinformatics technology and computer-aided drug design technology and further improve the ratio of positive charge and hydrophobicity through amino acid substitution and linking pheromones to construct AMPs with high antimicrobial activity and low toxicity (Yacoby and Benhar, 2007; Ong et al., 2014). Our study provides a method for constructing AMPs and screening out efficient, stable, and non-toxic AMPs, providing a solution for the creation of new aquatic antibiotic substitutes and the control of bacterial drug resistance and the large-scale spread of drug-resistant genes caused by the abuse of antibiotics.

## Materials and Methods

### Designing Antimicrobial Peptide-Derived Peptides

Previously, AMPs, mBjAMP1, have shown efficacy against Gram-negative *Escherichia coli* and *V. anguillarum* and Gram-positive *Staphylococcus aureus* and *M. luteus* (Liu et al., 2015). AMP1–6 was designed by replacing N^1^, A^4^, A^8^, and T^11^ of mBjAMP1 with W and replacing L^2^, C^3^, L^6^, R^7^, R^9^, R^20^, and R^21^ with K. The AMP consists of 21 amino acid residues, and after amino acid substitution, AMP1–6 was constructed. On this basis, we designed AMPs derived from heterozygosity by adding pheromones. We attempted to attach *Pseudomonas*-targeting fragments (Eckert et al., 2006), *S. aureus*-targeting fragments (Dunny and Leonard, 1997), and *L. monocytogenes* pheromones (Xayarath et al., 2015) to AMP1–6 to construct AMP-derived peptides P-6, S-6, and L-6, respectively.

**FIGURE 1:**
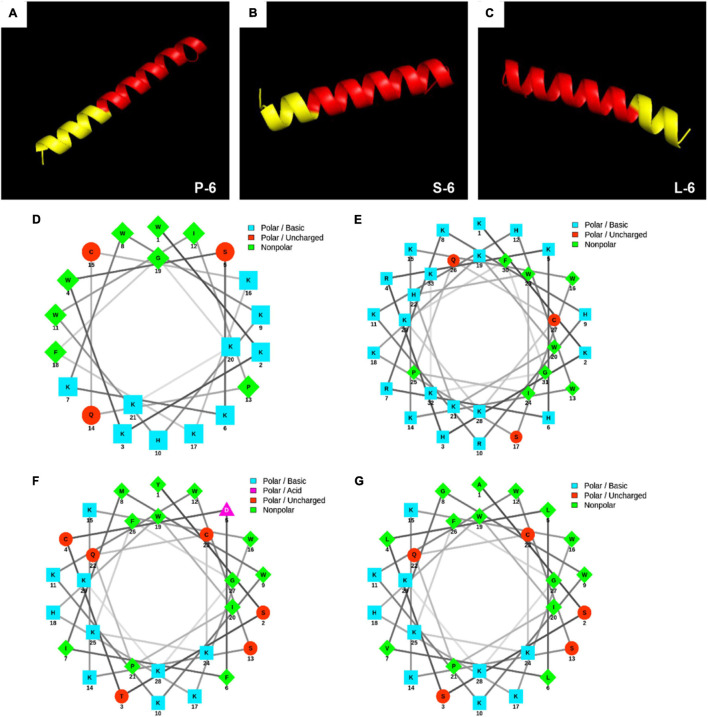
3D molecular modeling and helical wheel projections of antimicrobial peptides. 3D molecular modeling of P-6 **(A)**, S-6 **(B)**, and L-6 **(C)**. The red section represents antimicrobial peptides 1–6, and the yellow section represents the added targeted fragment. Helical wheel projections of 1–6 **(D)**, P-6 **(E)**, S-6 **(F)**, and L-6 **(G)**. In the spiral wheel projection, hydrophilic residues appear as circles, hydrophobic residues as diamonds, possibly negatively charged residues as triangles, and possibly positively charged residues as squares. Hydrophobicity is also indicated by color: the most hydrophobic residues are green, hydrophilic residues are red, positively charged ones are blue, and negatively charged ones are purple.

The physical and chemical properties of all AMPs were analyzed using a hydrophobic analysis website.^1^ The sequences and physicochemical properties of all AMPs are shown in Table 1. Antibacterial peptides were designed for three-dimensional structure modeling analysis using the 3D structure prediction website^2^ (Wang et al., 2018). The helical wheel projection of AMPs was calculated using the online program NetWheels: Peptides Helical Wheel and Net projection maker^3^ (Lv et al., 2014). The three-dimensional structure is shown in Figure 1.

**TABLE 1:** Amino acid sequences and chemical properties of all antimicrobial peptides.

Name	Sequence	Hydrophobic ratio	Net charge
mBjAMP1	NLCASLRARHTIPQ CKKFGRR	38%	+6
1–6	WKKWSKKWKHWIPQCK KFGKK	33%	+9
P-6	KKHRKHRKHRKHWKKWSKK WKHWIPQCKKFGKK	21%	+17
S-6	YSTCDFIMWKKWSKKWKHWI PQCKKFGKK	37%	+8
L-6	ASSLLLVGWKKWSKKWKHWI PQCKKFGKK	41%	+9

*The C-terminal residues of the peptides were amidated.*

*The added target fragments are underlined.*

### Peptide Synthesis and Bacterial Preparation

All AMPs were synthesized by GL Biochemical (Shanghai, China). Terminal amidation of AMPs was common, so all AMPs were modified with terminal amidation, and purity was greater than 95% (Lee et al., 1997). The peptides were dissolved in 1% (v/v) 2,2,2-trifluoroethanol water solution (2 mg/mL) and stored at −20°C until use.

The strains used in the experiment were Gram-negative *V. anguillarum*, *P. mendocina*, and *V. parahaemolyticus* and Gram-positive *M. luteus* and *L. monocytogenes*. *V. anguillarum* and *M. luteus* were preserved in the laboratory. *L. monocytogenes* CICC 21529 and *P. mendocina* CICC 21958 were obtained from the China Center of Industrial Culture Collection (CICC, China). *V. parahaemolyticus* (ATCC^®^17802^TM^) was obtained from the American Type Culture Collection. All strains were resuscitated by removing single colonies from an AGAR plate to a medium (Bengtsson et al., 2018). The bacteria were centrifuged at 5000 × *g* for 10 min, washed in phosphate-buffered saline (PBS) (pH 7.4), and resuspended in medium before use.

### Bactericidal Activity Assay

Bactericidal activity was assayed using the methods described by Bengtsson et al. (2018) with slight modifications. *V. anguillarum*, *M. luteus*, and *P. mendocina* were incubated in Luria-Bertani (LB) medium at 37°C for 16 h to the logarithmic growth period (Wang et al., 2018). *V. parahaemolyticus* was incubated in nutrient broth with 3% NaCl at 37°C for 16 h to the logarithmic growth period. *L. monocytogenes* was incubated in Brain Heart Infusion medium at 37°C for 16 h to the logarithmic growth period. The bacteria were flushed with PBS, and the bacterial concentration was adjusted to 4 × 10^4^ cells/mL. The final concentrations of the AMPs were 3, 6, 12, 25, and 50 μg/mL. The final volume in the Eppendorf tube was 100 μL. The concentration of AMPs was adjusted according to germicidal results, and PBS (pH 7.4) was used as the blank control. After incubation at 25°C for half an hour, the protein-bacterial mixture of each concentration was divided into three parts on average, coated on three glass plates containing LB solid medium, and cultured overnight at 37°C. The colony count method was used to count the number of colonies on each plate and calculate the fungicidal rate of proteins. The minimum bactericidal concentration (MBC) is defined as the minimum drug concentration required to kill 99.9% of bacteria (Malanovic et al., 2020).

$$\mathrm{Bactericidal}\,\mathrm{activity}=\left(\frac{\begin{array}{c} \mathrm{bacterial}\,\mathrm{colony}\,\mathrm{number}\,\mathrm{of}\,\mathrm{control}\,\mathrm{group}\;\\ -\mathrm{bacterial}\,\mathrm{colony}\,\mathrm{number}\,\mathrm{of}\,\mathrm{experimental}\,\mathrm{group}\; \end{array}}{\mathrm{bacterial}\,\mathrm{colony}\,\mathrm{number}\,\mathrm{of}\,\mathrm{control}\,\mathrm{group}}\right)\times 100\%$$

### Antibacterial Activity Assay

The antibacterial activity of the synthetic AMPs was tested using *P. mendocina*, *L. monocytogenes*, and *V. parahaemolyticus*. The antimicrobial activity was measured as described by Yang et al. (2006) with slight modifications. The conditions for culture and recovery of *P. mendocina*, *L. monocytogenes*, and *V. parahaemolyticus* were the same as above. The bacteria were flushed with PBS, and the bacterial concentration was adjusted to 10^6^ cells/mL for subsequent experiments. The liquid medium, diluted bacteria, and antibacterial peptides of different concentrations (3, 5, 7.5, 8.5, 10, and 12.5 μg/mL) were mixed and added into a 96-well plate (200 μL per well), and the mixture was cultured for 8 h. PBS (pH 7.4) was used as the negative control, and the antibiotics kanamycin sulfate and ampicillin sodium were used as positive controls. During this period, the absorbance of the 595 nm wavelength was measured using a Multiskan MK3 microplate reader (Thermo Fisher Scientific, China) every hour. According to the results, the growth curve of the bacteria was drawn, and the minimum inhibitory concentration (MIC) was defined as the lowest AMP concentration at which the growth of the respective bacteria was completely inhibited (Wang et al., 2018). The MICs of each AMP against different bacteria were also calculated.

### Transmission Electron Microscopy

The morphological changes in bacteria treated with AMPs were observed under a transmission electron microscope (TEM). The sample treatment method was slightly modified according to the experimental method described by Lv et al. (2014). *V. anguillarum*, *M. luteus*, and *L. monocytogenes* CICC 21529 were centrifuged at 25°C at 5000 × *g* for 3 min, the liquid supernatant was removed, and PBS was used to wash the bacteria separately. This procedure was repeated three times, and bacteria were finally resuspended at a concentration of 10^9^ cells/mL. The bacterial solution was mixed with the peptide solution, and the final concentration of the AMP was set to 25 μg/mL, with a final volume of 100 μL. PBS was used as the blank control. After incubation of AMPs and bacteria at 25°C for 30 min, 2.5% glutaraldehyde (prepared with PBS, pH 7.4) solution of the same volume was added to the mixture for 10 min to fix the bacterial cells. The prepared samples were fixed in 50 μL 2.5% glutaraldehyde solution (Sigma-Aldrich, G5882). Then, they were dropped onto 400-mesh carbon-coated grids and allowed to stand at room temperature for 3 min. Excess fluid was removed by touching the edge of a filter paper. The grids were then dried using the filter paper. The sample preparation method of the *M. luteus* ultrathin section is the same as the above. The incubated mixture was fixed with 2.5% glutaraldehyde, dehydrated with acetone gradient, embedded with Epon812, and cut into ultrathin sections with a glass knife on a microtome, stained with uranyl acetate-lead citrate. Observations were performed using a JEOL JSM-840 TEM.

### Membrane Depolarization Assay

The membrane depolarization activity of 1–6, P-6, S-6, and L-6 were assayed using the membrane potential-sensitive dye DiSC_3_-5 (Sigma-Aldrich). *V. anguillarum*, *M. luteus*, *L. monocytogenes*, *P. mendocina*, and *V. parahaemolyticus* were cultured to the logarithmic growth period and centrifuged at room temperature (6000 × *g* for 5 min), the upper medium was removed, and the bacteria were washed with 5 mM 2-[4-(2-hydroxyethyl)-1-piperazinyl] (HEPES) buffer. This procedure was repeated thrice, and bacteria were resuspended in HEPES buffer containing 2 mM ethylenediaminetetraacetic acid (EDTA) and 100 mM KCl. The bacterial concentration was adjusted to that of approximately 0.05 OD600, for subsequent experiments. Aliquots of bacterial suspensions (100 μL) were transferred to each well of a 96-well flat-bottom white microplate, and a stock solution of DiSC_3_-5 was added to the bacterial suspensions, yielding a final concentration of 0.5 μM. After incubation for 30 min in the dark, DISC_3_-5 could be fully absorbed and reached a stable reference value for fluorescence. The bacterial suspensions were then mixed with 100 μL of different concentrations of AMPs 1–6, P-6, S-6, and L-6, giving the desired concentrations of 30 μg/mL and 50 μg/mL. HEPES buffer (5 mM) was used as the blank control. Changes in fluorescence intensity were continuously recorded for 30 min with a fluorimeter at an excitation wavelength of 622 nm and an emission wavelength of 670 nm.

### Membrane Permeability Assay

The bacterial membrane provides excellent protection (Klubthawee et al., 2020). To explore the effect of AMPs on the permeability of bacterial cell membranes, a fluorescent dye, propidium iodide (PI, Solarbio), which can bind to DNA, was used in this study (Alvarez-Barrientos et al., 2000). *V. anguillarum*, *M. luteus*, *L. monocytogenes*, *P. mendocina*, and *V. parahaemolyticus* were centrifuged at room temperature at 5000 × *g* for 3 min, the liquid supernatant was removed, and PBS (pH 7.4) was used to wash the bacteria separately. This procedure was repeated three times (Klubthawee et al., 2020). Finally, the bacteria were resuspended to adjust the concentration to 10^8^ cells/mL. The AMPs were mixed with the bacterial solution, and final concentrations of 12.5 and 25 μg/mL were achieved, with a final volume of 600 μL. PBS (pH 7.4) was used as a blank control. After incubation at room temperature for 30 min, 0.75 μL of 5 mg/mL PI was added and incubated at 4°C in the dark for 1 h. The bacterial cells stained with PI were examined using an FC500 MPL flow cytometer (Beckman), and the data were analyzed using WinMDI v.2.9 software (Scripps Research Institute, San Diego, CA, United States).

### Reactive Oxygen Species Assay

The levels of reactive oxygen species (ROS) were measured based on the intracellular peroxide-dependent oxidation of 2′, 7′-dichlorofluorescein diacetate (DCFH2-DA), a non-fluorescent lipophilic probe that can cross the cell membrane. Inside the cell, DCFH2-DA deacetylates to form DCFH2, which is also a non-fluorescent probe that cannot diffuse freely across the cell membrane. DCFH2 reacts with intracellular ROS to yield the highly fluorescent compound 2′, 7′-dichlorofluorescein (DCF), which can reflect the intracellular levels of ROS. A ROS detection kit was used following the manufacturer’s instructions (Nanjing Jiancheng Bioengineering Institute, China). The bacteria were cultured to mid-logarithmic phase and resuspended in LB medium containing 10 mM DCFH2-DA, yielding a density of 10^7^ cells/mL. After incubation at 37°C for 30 min, the bacteria were collected by centrifugation at 6000 × *g* at 25°C for 10 min. Probes that did not enter bacterial cells were removed. The bacterial cells were washed in PBS (pH 7.4) three times, added to 1–6, P-6, S-6, or L-6, and resuspended at a final concentration of 12.5 μg/mL. For the positive control, bacterial cells were resuspended in 1 mL containing 90 μmol/L H_2_O_2_ (a compound mixture that can significantly increase ROS levels in cells within 30 min) (Nanjing Jiancheng Bioengineering Institute, China). For the blank control, cells were resuspended in 1 mL PBS alone. The bacterial suspensions were incubated at 25°C for 1 h, and the fluorescence intensities were recorded immediately using a spectrofluorometer at excitation and emission wavelengths of 488 and 525 nm, respectively.

### 3-(4,5-Dimethyl-2-Thiazolyl)-2,5-Diphenyl-2H-Tetrazolium Bromide Assay

To determine whether AMPs are cytotoxic to murine RAW264.7, a 3-(4,5-dimethyl-2-thiazolyl)-2,5-diphenyl-2H-tetrazolium bromide (MTT) assay was performed as described by Cui et al. (2016). The MTT assay can be used to detect cellular activity (Liu et al., 2017). RAW264.7 cells were suspended in serum-free DMEM, and 100 μL cell suspension (1 × 10^6^ cells/mL) was placed in a 96-well plate and cultured at 37°C with 5% CO_2_ for 2 h. AMPs were added, producing final concentrations of 12.5, 25, and 50 μg/mL. The final volume of the 96-well plate was 200 μL, followed by incubation for 4 h. PBS (pH 7.4) was used as a blank control. MTT (20 μL, 5 mg/mL) dissolved in PBS was added to each well, followed by incubation for 4 h. The liquid in each well was removed, 150 μL dimethyl sulfoxide (DMSO) was added, and the absorbance value of the sample was measured at a wavelength of 492 nm with a Multiskan MK3 microplate reader (Thermo Fisher Scientific, China). The percent viability against the control was calculated as follows: (OD of treated group/OD of control group) × 100% (*n* = 3).

### Fish Maintenance and Life-Cycle Exposure

All experimental procedures complied with the National Institute of Health Guide for the Care and Use of Laboratory Animals and were approved by the Animal Care Committee of Ocean University of China. Ice bath anesthesia was used for all experiments. *Sebastes schlegelii* kept under optimal conditions [pH 7.8 ± 0.2,25°C, 14 h (light):10 h (darkness)] and feeding once a day. Renew the artificial seawater every morning before feeding. Before the experiment, the 2-year-old *S. schlegelii* was randomly collected and domesticated for a week. Eighty healthy adult fish with an average body weight of 7 g were randomly divided into four groups with 15 fish in each group. Each group was maintained in glass jars with 50 L of artificial seawater at a salinity of 30‰. In order to detect the effects of AMPs and antibiotics on the intestinal flora of fish. The fish were fed with 1–6, L-6 and Enrofloxacin at 5 mg/mL for 7 days, and each fish was fed with 10 μL per day. The blank control group did not receive any treatment. All *S. schlegelii* were dissected and the intestines were collected. Intestines of three individuals of the same were pooled as a replicate. There were three replicates for each exposure group (*n* = 5), which were rapidly frozen in liquid nitrogen and then stored at −80°C for further analysis. All experimental procedures complied with the National Institute of Health Guide for the Care and the Use of Laboratory Animals and were approved by the Animal Care Committee of Ocean University of China. The exposure experiments were performed in semistatic conditions according to OECD Guidelines 210.35 Ice bath anesthesia was used for all experiments.

### DNA Extraction and 16S rRNA Amplicon Sequencing

DNA was extracted from 2.10 intestinal samples using the QIAamp DNA Mini Kit (QIAGEN, Germany). The DNA concentration was validated with NanoDrop and agarose gel to ensure that the quality of the extracted DNA met the requirements for subsequent amplification. According to 16S rRNA gene sequence characteristics and MiSeq Platform sequencing requirements, designed specific primer 338F for V3–V4 region: 5′-ACTCCTACGGGAGGCAGCA-3′, 806R: 5′-GGACTACHVGGGTWTCTAAT-3′ was amplified by PCR. After PCR product quantification and homogenization, MiSeq platform was used for sequencing analysis by OE Biotech Co., Ltd. (Shanghai, China). The library sequencing and data processing were conducted by OE biotech Co., Ltd. (Shanghai, China).

### Statistical Analysis

All experiments were performed in triplicates and repeated three times, except for the TEM experiment. Statistical analyses were performed using GraphPad Prism version 5. All data are expressed as means ± standard deviation. The significance of differences was determined using two-way ANOVA, and differences were considered statistically significant at values of *p* < 0.05.

## Results

### Characteristics of Synthesized Peptides

As shown in Figure 1, all peptides have distinct hydrophobic and hydrophilic regions, which make them amphiphilic. Using the 3D structure prediction website, 3D structure modeling analysis of the designed AMPs showed that they all had an α-helical structure. This three-dimensional structure plays an important role in the binding of AMPs to microorganisms. The hydrophobic site analysis showed that the net charge range of all peptides was +8 to +17 (Table 1). The helical wheel projection of the peptides shown in Figures 1D–F illustrates the assembly of hydrophobic residues WLAW in a single face, which is significant for membranolytic activity.

### Bactericidal Activity

**FIGURE 2:**
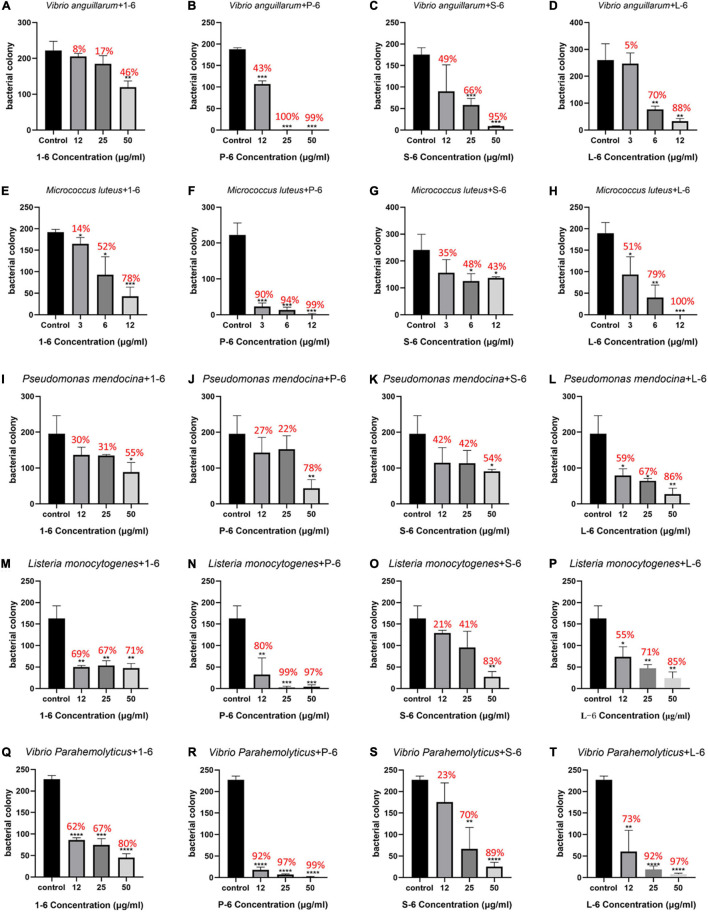
Bactericidal activities of 1–6, P-6, S-6, and L-6 against five bacteria. **(A)** *V. anguillarum* treated with 1–6. **(B)** *V. anguillarum* treated with P-6. **(C)** *V. anguillarum* treated with S-6. **(D)** *V. anguillarum* treated with L-6. **(E)** *M. luteus* treated with 1–6. **(F)** *M. luteus* treated with P-6. **(G)** *M. luteus* treated with S-6. **(H)** *M. luteus* treated with L-6. **(I)** *P. mendocina* treated with 1–6. **(J)** *P. mendocina* treated with P-6. **(K)** *P. mendocina* treated with S-6. **(L)** *P. mendocina* treated with L-6. **(M)** *L. monocytogenes* treated with 1–6. **(N)** *L. monocytogenes* treated with P-6. **(O)** *L. monocytogenes* treated with S-6. **(P)** *L. monocytogenes* treated with L-6. **(Q)** *V. parahaemolyticus* treated with 1–6. **(R)** *V. parahaemolyticus* treated with P-6. **(S)** *V. parahaemolyticus* treated with S-6. **(T)** *V. parahaemolyticus* treated with L-6. All data are expressed as means ± SD. The *t*-test was used to evaluate significant differences between controls and treatment groups. *p < 0.05, **p < 0.01, ***p < 0.001, and ****p < 0.0001.

First, the bactericidal activity of the designed AMPs was studied. It was found that AMP1–6 was capable of significantly killing *V. anguillarum*, *M. luteus*, *P. mendocina*, *L. monocytogenes*, and *V. parahaemolyticus* at the concentration tested (50 μg/mL), with bactericidal rates of 46, 78, 55, 71, and 80%, respectively (Figures 2A,E,I,M,Q). S-6 killed *V. anguillarum*, *M. luteus*, *P. mendocina*, *L. monocytogenes*, and *V. parahaemolyticus* at the concentration tested (50 μg/mL), with bactericidal rates of 95, 43, 54, 83, and 89%, respectively (Figures 2C,G,K,O,S). L-6 killed *V. anguillarum*, *M. luteus*, *P. mendocina*, *L. monocytogenes*, and *V. parahaemolyticus* at the concentration tested (50 μg/mL), with bactericidal rates of 88, 100, 86, 85, and 97%, respectively (Figures 2D,H,L,P,T). P-6 displayed higher rates of killing *V. anguillarum*, *M. luteus*, *P. mendocina*, *L. monocytogenes*, and *V. parahaemolyticus* at the concentration tested (50 μg/mL), with bactericidal rates of 99, 99, 78, 97, and 99%, respectively (Figures 2B,F,J,N,R). Compared with AMP1–6, AMPs P-6, S-6, and L-6 with pheromone fragments significantly increased the bactericide rate against *V. anguillarum*, *P. mendocina*, and *V. parahaemolyticus* at the concentrations tested (12, 25, and 50 μg/mL). AMP P-6 (25 μg/mL) had a 99% bactericidal rate against *V. anguillarum*. The AMPs P-6 and L-6 (12 μg/mL) demonstrated a 99% bactericidal rate against *M. luteus*. The MBCs are shown in Table 2. In conclusion, compared with mBjAMP1 without bactericidal activity, AMP1–6 and AMPs with pheromones display broad-spectrum bactericidal activity, and AMPs with pheromones had a stronger bactericidal effect than AMP1–6.

**TABLE 2:** Minimum bactericidal concentration (MBC) of four antimicrobial peptides against bacteria.

Peptides	MBC (μg/mL)				
	*V. anguillarum*	*M. luteus*	*L. monocytogenes*	*P. mendocina*	*V. parahaemolyticus*
1–6	>50	>12	>50	>50	>50
*P*-6	25	12	25	>50	50
*S*-6	>50	>12	>50	>50	>50
*L*-6	>12	12	>50	>50	>50

### Antibacterial Activity

**FIGURE 3:**
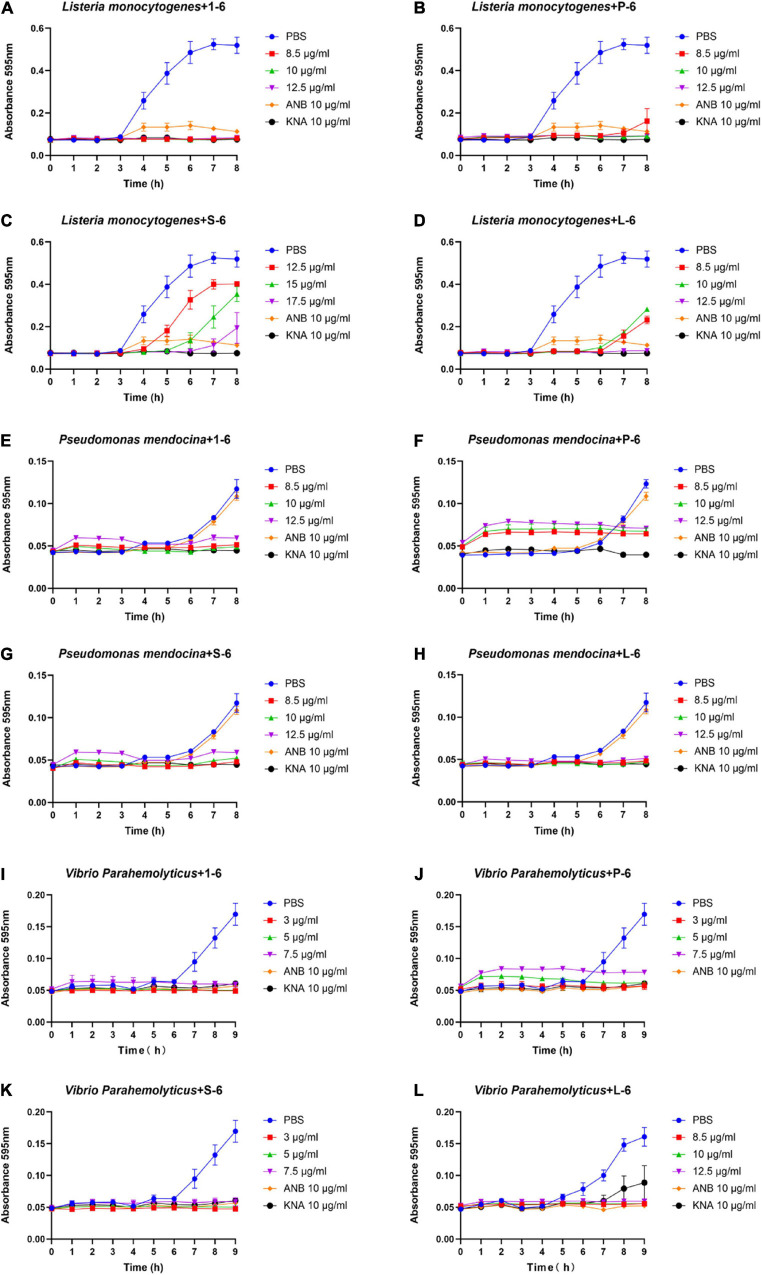
Antibacterial activities of 1–6, P-6, S-6, and L-6 against three bacteria. **(A)** *L. monocytogenes* treated with 1–6. **(B)** *L. monocytogenes* treated with P-6. **(C)** *L. monocytogenes* treated with S-6. **(D)** *L. monocytogenes* treated with L-6. **(E)** *P. mendocina* treated with 1–6. **(F)** *P. mendocina* treated with P-6. **(G)** *P. mendocina* treated with S-6. **(H)** *P. mendocina* treated with L-6. **(I)** *V. parahaemolyticus* treated with 1–6. **(J)** *V. parahaemolyticus* treated with P-6. **(K)** *V. parahaemolyticus* treated with S-6. **(L**) *V. parahaemolyticus* treated with L-6.

As a positive control, ampicillin sodium had no inhibitory effect on *P. mendocina*. The growth of all three strains of bacteria was inhibited by AMP1–6, P-6, S-6, and L-6 (Figure 3), with MICs ranging from 1.077 to 3.051 μM, 0.675 to 2.250 μM, 0.800 to 4.669 μM, and 2.410 to 3.544 μM, respectively (Table 3). mBjAMP1 has an MIC range of 2.565–5.090 μM against several bacteria. The antimicrobial activity was improved compared with that of mBjAMP1 (Liu et al., 2015). The antimicrobial activity of AMPs with added pheromones was also enhanced, compared with that of AMP1–6. The antimicrobial activity of P-6, S-6, and L-6 against *P. mendocina* was increased, and the antimicrobial activity of P-6 and S-6 against *V. parahaemolyticus* was increased, compared with that of AMP1–6. In conclusion, these results showed that AMP1–6, P-6, S-6, and L-6 effectively inhibited bacterial growth.

**TABLE 3:** Minimum inhibitory concentrations (MICs) of four antimicrobial peptides against bacteria.

Peptides	MIC (μM)		
	*L. monocytogenes*	*P. mendocina*	*V. parahaemolyticus*
1–6	3.051	3.051	1.077
P-6	2.250	1.913	0.675
S-6	4.669	2.268	0.800
L-6	3.544	2.410	<2.410

### Destruction of Bacterial Cells by Antimicrobial Peptides

**FIGURE 4:**
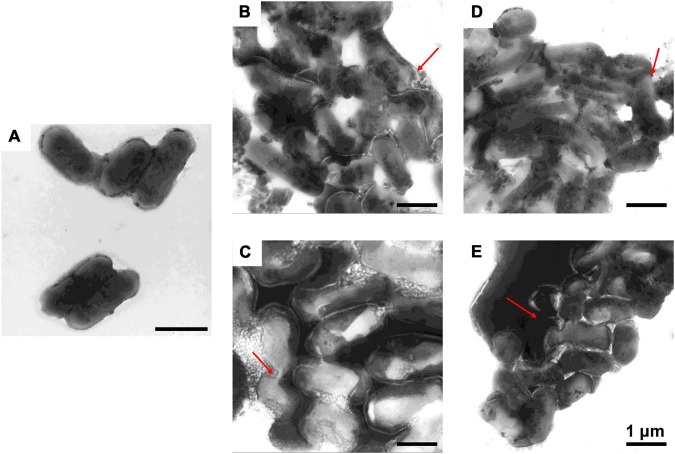
Transmission electron microscopic observation of *V. anguillarum*. *V. anguillarum* incubated with PBS **(A)** or with 1–6, P-6, S-6, and L-6 **(B–E)** at 28°C for 30 min. The red arrow points to where the cell membrane has broken and the cell contents have flowed out.

**FIGURE 5:**
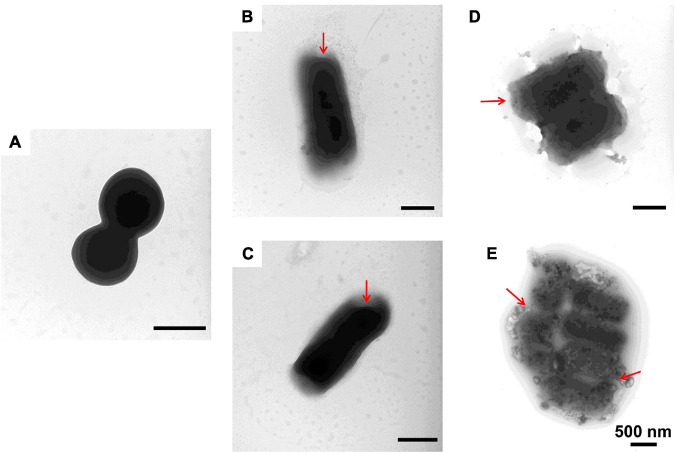
Transmission electron microscopic observation of *M. luteus*. *M. luteus* incubated with PBS **(A)** or with 1–6, P-6, S-6, and L-6 **(B–E)** at 28°C for 30 min. The red arrow points to where the cell membrane has broken and the cell contents have flowed out.

**FIGURE 6:**
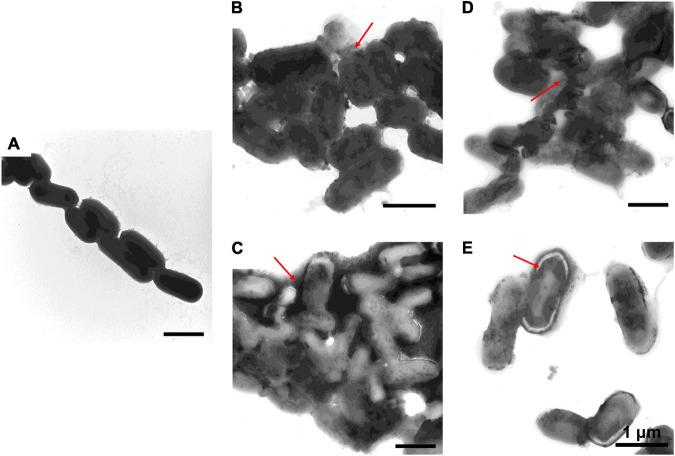
Transmission electron microscopic observation of *L. monocytogenes*. *L. monocytogenes* incubated with PBS **(A)** or with 1–6, P-6, S-6, and L-6 **(B–E)** at 28°C for 30 min. The red arrow points to where the cell membrane has broken and the cell contents have flowed out.

To further study the effect of AMPs on bacterial cell structure, *V. anguillarum*, *M. luteus*, and *L. monocytogenes* were incubated with the designed AMPs and observed using a TEM. The results of TEM analysis showed that, compared with the control group (Figures 4A, 5A, 6A), the bacterial shape changed obviously after incubation with AMPs for 0.5 h, and the bacterial shape became irregular (Figures 4B–E, 5B–E), intracellular contents flowed out of cells (Figures 5E, 6B–E), and holes appeared on the surfaces of some bacterial cells. At the same time, we observed the ultrathin section of *M. luteus* by electron microscope (Supplementary Figure 14). The cell wall and cell membrane of *M. luteus* were wrinkled and damaged after incubated with AMP, which further confirmed the destructive effect of AMP on bacteria. It was found that AMP1–6, P-6, S-6, and L-6 caused direct damage to *V. anguillarum*, *M. luteus*, and *L. monocytogenes*, resulting in membrane disruption. Cytoplasmic leakage was also observed in bacteria treated with AMP1–6, P-6, S-6, and L-6. These results showed that AMP1–6, P-6, S-6, and L-6 are bactericidal agents capable of directly killing *V. anguillarum*, *M. luteus*, and *L. monocytogenes*.

### Membranolytic Activity of Peptides Toward Bacteria

**FIGURE 7:**
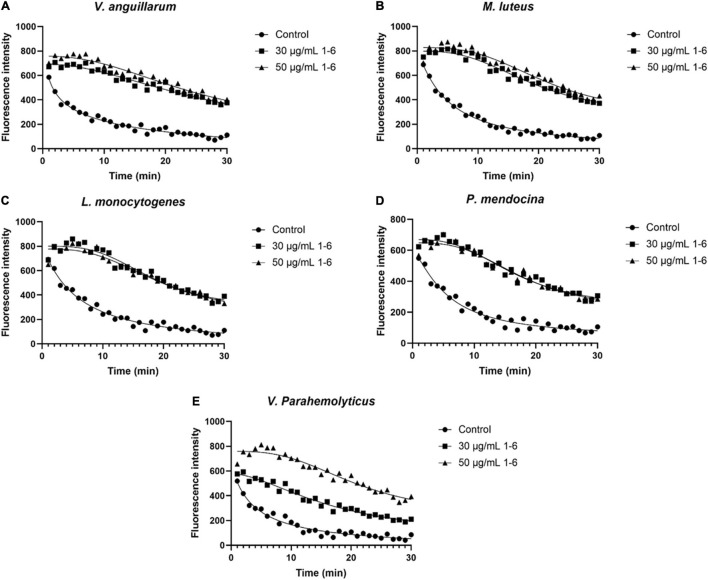
Bacterial membrane depolarization. Depolarization of the bacterial membrane after incubation with antimicrobial peptides 1–6 was detected using DiSC_3_-5 (excitation, 622 nm; emission, 670 nm). **(A)** Depolarization of cell membrane of *V. anguillarum* incubated with antimicrobial peptides 1–6. **(B)** Depolarization of cell membrane of *M. luteus* incubated with antimicrobial peptides 1–6. **(C)** Depolarization of cell membrane of *L. monocytogenes* incubated with antimicrobial peptides 1–6. **(D)** Depolarization of cell membrane of *P. mendocina* incubated with antimicrobial peptides 1–6. **(E)** Depolarization of cell membrane of *V. parahaemolyticus* incubated with antimicrobial peptides 1–6.

The membrane depolarization activities of AMP1–6, P-6, S-6, and L-6 were assayed using DiSC_3_-5. As shown in Figures 7A–E (Supplementary Figures 1–3), the fluorescence intensity of five bacterial cells treated with AMP1–6, P-6, S-6, and L-6 increased significantly compared with that of controls, indicating that AMP1–6, P-6, S-6, and L-6 caused depolarization of the bacterial plasma membrane. This indicated that AMP1–6, P-6, S-6, and L-6 can act on the cell membrane and alter membrane potential, thus affecting the physiological function of bacteria.

**FIGURE 8:**
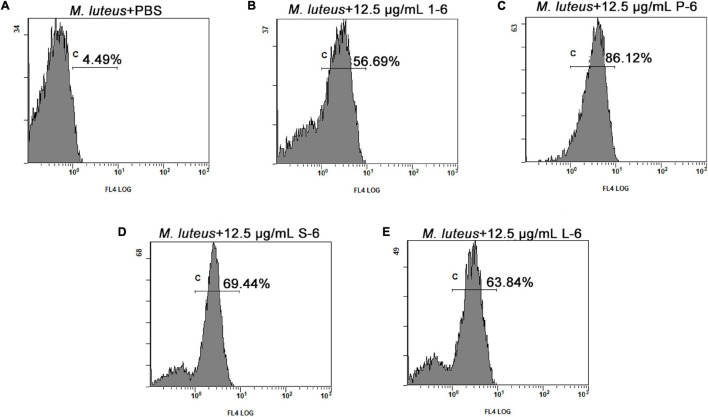
Membrane permeation of *M. luteus*. The effects of 12.5 μg/ml of 1–6, P-6, S-6, and L-6 on the membrane integrity of *M. luteus* cells were analyzed using flow cytometry. The proportion of permeabilized cells is shown near c. **(A)** The effects of 12.5 μg/ml of PBS on the membrane integrity of *M. luteus* cells were analyzed using flow cytometry. **(B)** The effects of 12.5 μg/ml of 1–6 on the membrane integrity of *M. luteus* cells were analyzed using flow cytometry. **(C)** The effects of 12.5 μg/ml of P-6 on the membrane integrity of *M. luteus* cells were analyzed using flow cytometry. **(D)** The effects of 12.5 μg/ml of S-6 on the membrane integrity of *M. luteus* cells were analyzed using flow cytometry. **(E)** The effects of 12.5 μg/ml of L-6 on the membrane integrity of *M. luteus* cells were analyzed using flow cytometry.

The proportion of fluorescent bacteria detected using flow cytometry reflects the proportion of changes in bacterial cell permeability. Bacteria treated with AMPs showed different proportions of PI fluorescence. As shown in Figure 8 (Supplementary Figures 4–12), a significant proportion of bacterial cells treated with AMP1–6, P-6, S-6, and L-6 displayed PI fluorescent signals, indicating that the membranes of the bacterial cells were permeabilized by AMP1–6, P-6, S-6, and L-6. By contrast, few cells in untreated bacteria showed PI fluorescent signals, indicating that their cell membranes were intact and functional. These data indicate that the designed AMPs can destroy the membrane integrity of bacterial cells.

### Antimicrobial Peptides 1–6, P-6, S-6, and L-6 Increase Intracellular Reactive Oxygen Species Level

**FIGURE 9:**
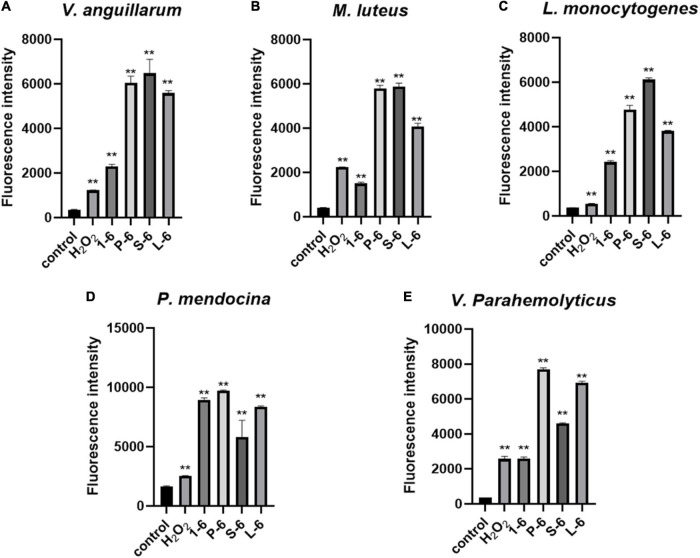
Effects of 1–6, P-6, S-6, and L-6 on intracellular ROS levels. **(A)** *V. anguillarum* treated with 1–6, P-6, S-6, and L-6. **(B)** *M. luteus* treated with 1–6, P-6, S-6, and L-6. **(C)** *L. monocytogenes* treated with 1–6, P-6, S-6, and L-6. **(D)** *P. mendocina* treated with 1–6, P-6, S-6, and L-6. **(E)** *V. parahaemolyticus* treated with 1–6, P-6, S-6, and L-6. H_2_O_2_, a compound mixture, significantly increased ROS levels in cells within 30 min. The *t*-test was used to evaluate significant differences between controls and treatment groups. **p < 0.01.

High levels of intracellular ROS can cause necrosis and apoptosis. When *V. anguillarum*, *M. luteus*, *L. monocytogenes*, *P. mendocina*, and *V. parahaemolyticus* cells were treated with AMP1–6, P-6, S-6, or L-6, their intracellular ROS levels increased significantly (Figure 9). These results showed that AMP1–6, P-6, S-6, and L-6 induced apoptosis/necrosis of bacterial cells.

### Non-cytotoxicity of Peptides to Mammalian Cells

**FIGURE 10:**
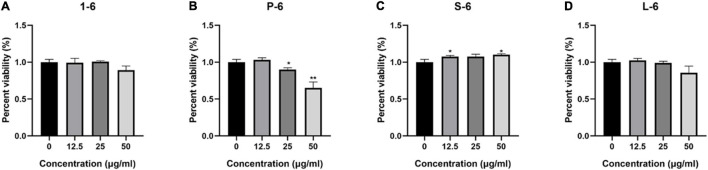
The percent viability of RAW264.7 cells. The percent viability of RAW264.7 cells in the presence of 1–6 **(A)**, P-6 **(B)**, S-6 **(C)**, and L-6 **(D)**. The *t*-test was used to evaluate significant differences between controls and treatment groups. *p < 0.05, **p < 0.01.

The MTT assay was used to determine whether four AMPs were toxic to mammalian cells. These data indicated that AMP1–6, P-6, S-6, and L-6 had little or no cytotoxic effect on mammalian cells, indicating that AMPs have a preference for bacterial cell membranes (Figure 10).

### Analysis of Intestinal Microflora of *Sebastes schlegelii*

**FIGURE 11:**
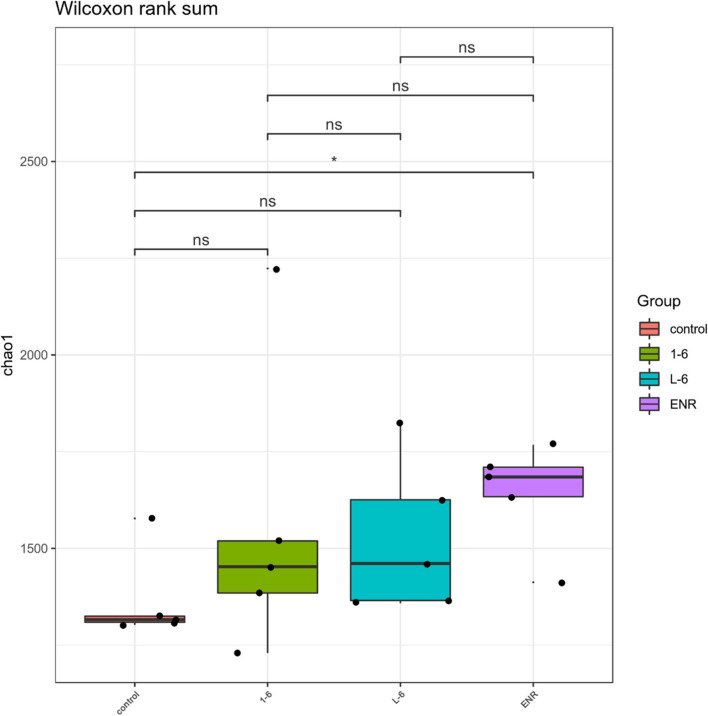
Analysis of intestinal microflora α diversity of *Sebastes schlegelii*. The *t*-test was used to evaluate significant differences between controls and treatment groups. *p < 0.05.

**FIGURE 12:**
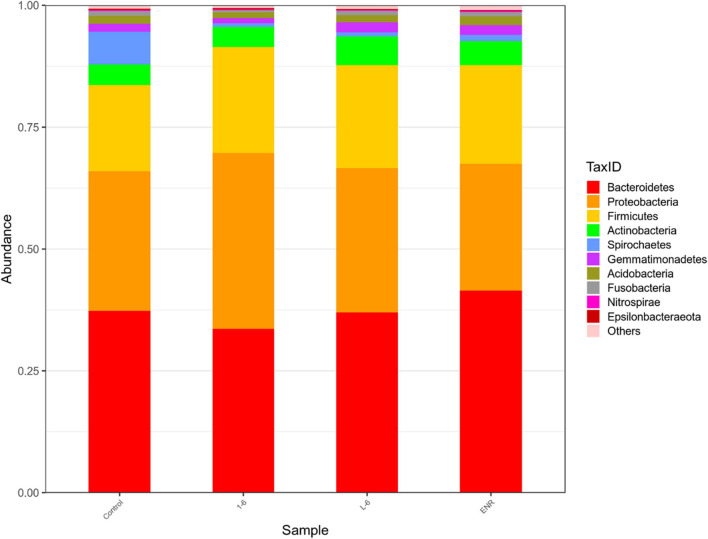
Structure and composition of intestinal microflora of *Sebastes schlegelii* at phylum level. The relative abundance of *Bacteroides* and *Proteobacteria* in the control group was 37.24 and 28.53%, respectively. The relative abundance of *Bacteroides* and *Proteobacteria* in 1–6 group was 34.03 and 36.40%, respectively. The relative abundance of *Bacteroides* and *Proteobacteria* in L-6 group was 37.13 and 29.58%, respectively. The relative abundance of *Bacteroides* and *Proteobacteria* in enrofloxacin group was 41.47 and 25.97%, respectively.

**FIGURE 13:**
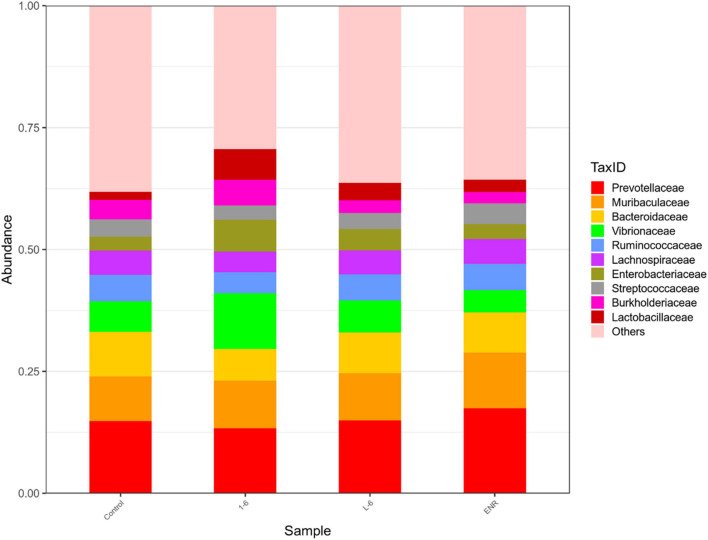
Structure and composition of intestinal microflora of *Sebastes schlegelii* at family level. The relative abundance of *Prevotellaceae*, *Muribaculaceae*, *Bacteroidaceae*, *Lactobacillaceae*, and *Streptococcaceae* in the control group were 14.84, 9.1, 9.1, 1.7, and 3.5%, respectively. The relative abundance of *Prevotellaceae*, *Bacteroidaceae*, and *Lactobacillaceae* in 1–6 groups were 13.44, 6.63, and 5.64%, respectively. The relative abundance of *Lactobacillaceae* in L-6 group was 3.57%. The relative abundance of *Prevotellaceae*, *Streptococcaceae*, and *Muribaculaceae* in enrofloxacin group were 17.41, 4.32, and 11.36%, respectively.

As shown in Figure 11, the α diversity of intestinal microflora in *S. schlegelii* was significantly increased after enrofloxacin (ENR) treatment, while there was no significant change in the diversity of intestinal microflora after 1–6 and L-6 treatment. At the phylum level, the eight dominant bacterial groups in all treatments were *Bacteroides*, *Proteobacteria*, *Firmicutes*, *Actinobacteria*, *Spirochaetes*, and *Gemmatimonadetes* which together accounted for >90% of the total gut microbiome abundance. At phylum level, the relative abundance of *Bacteroides* and *Proteobacteria* in the control group was 37.24 and 28.53% respectively. The relative abundance of *Bacteroidetes* and *Proteobacteria* in enrofloxacin group increased by 41.74% and decreased by 25.97%, respectively (Figure 12). The relative abundance of *Bacteroidetes* in groups 1–6 and L-6 was slightly decreased 34.03 and 37.13% compared with the control group, respectively. The relative abundance of *Proteobacteria* in groups 1–6 and L-6 was increased by 36.40 and 29.58% compared with the control group, respectively (Figure 12). At the Family level, the relative abundance of *Prevotellaceae*, *Muribaculaceae*, *Bacteroidaceae*, *Lactobacillaceae*, and *Streptococcaceae* in the control group was 14.84, 9.1, 9.1, 1.7, and 3.5% respectively. Compare with the control group, the relative abundance of *Prevotellaceae* (13.44%) and *Bacteroidaceae* (6.63%) decreased slightly in 1–6 groups, while the relative abundance of *Lactobacillaceae* (5.64%) increased. The relative abundance of bacteria in L-6 group was similar to that in the control group, and the relative abundance of *Lactobacillaceae* (3.57%) increased. In enrofloxacin group, the relative abundance of *Prevotellaceae* (17.41%), *Streptococcaceae* (4.32%), and *Muribaculaceae* (11.36%) increased (Figure 13).

## Discussion

In aquaculture, diseases caused by aquatic pathogenic bacteria cause huge economic losses. As antibiotics act on specific targets, bacteria are prone to develop resistance mechanisms (Zgurskaya et al., 2015). Therefore, to eliminate the consequences of antibiotic abuse, it is imperative to develop new antimicrobial agents (Naylor et al., 2009). AMPs act directly on bacterial cell membranes and are unlikely to produce resistance mechanisms; therefore, they are expected to replace antibiotics (Boman, 2003). However, natural AMPs have certain drawbacks such as low antimicrobial effects and high production costs. The electrostatic interaction between the negatively charged membrane and the cationic peptide plays a key role in the formation of the initial complex between AMP and the membrane (Yeaman and Yount, 2003). Therefore, positively charged amino acid residues are of particular interest for AMPs. Previously, we proved that mBjAMP1 exerts antibacterial effects on *E. coli*, but the antibacterial effect is weak, and no bactericidal effect was observed (Liu et al., 2015). Tryptophan (W) residues are known for good hydrophobic interactions in the membrane (Petersen et al., 2005; Chan et al., 2006), and lysine (K) residues are used to increase the cationic properties of AMPs and promote interactions with the target cell surface (Park et al., 2009). Therefore, we constructed AMP1–6 by replacing N^1^, A^4^, A^8^, and T^11^ of mBjAMP1 with W, and replacing L^2^, C^3^, L^6^, R^7^, R^9^, R^20^, and R^21^ with K. Based on this, we added pheromones at the N-terminus to further evaluate the antibacterial and toxic effects of the designed AMP. We attempted to attach *Pseudomonas*-targeting fragments (Eckert et al., 2006), *S. aureus*-targeting fragments (Dunny and Leonard, 1997), and *L. monocytogenes* pheromones (Xayarath et al., 2015) to AMP1–6 to construct AMPs P-6, S-6, and L-6, respectively. The 3D structure modeling analysis of the AMPs was conducted using a 3D structure prediction website and revealed that all AMPs displayed an amphiphilic α-helical structure, which is a common structure in the existing AMPs. The number of amino acids in the designed AMPs was between 21 and 33, and this short peptide greatly reduced the production cost.

All four peptides increased their positive charge, and L-6 increased the proportion of hydrophobicity (Table 1). Peptides have lysine residues that contribute to the stability of their secondary structures (Torres et al., 2019). Moreover, peptides can be inserted into the hydrophobic core of anionic membranes to improve their antimicrobial activity (Singh et al., 2016). Giangaspero et al. (2001) showed that maximum antimicrobial efficacy can be obtained when high charges (cationic) and amphiphilicity are present. Our experimental results confirmed this point. All designed peptides were more effective against both Gram-positive and Gram-negative bacteria than mBjAMP1, which had no bactericidal activity. Studies have shown that a water-soluble lectin isolated from *Moringa oleifera* seeds (WSMoL) has no bactericidal effect on *M. luteus* (Coriolano et al., 2020). The MBC of L-6, with the highest proportion of hydrophobic residues, was the smallest for the aquatic pathogenic bacteria *V. anguillarum* and *M. luteus*. For *L. monocytogenes*, *P. mendocina*, and *V. parahaemolyticus*, all AMPs exhibited ideal bacteriostatic effects, with MICs lower than that of common antibiotics and most other AMPs (Li et al., 2016; Table 4). P-6, with the highest positive charge, displayed the best bacteriostatic effect and the lowest MIC value. Zhou et al. (2020) showed that the MIC of the mature peptide of PaBD (mPaBD) had no inhibitory effect on *V. parahaemolyticus*. Yang et al. (2020) showed that MICs of the AMP TGH1 against *V. parahaemolyticus* and *L. monocytogenes* were 6.1 and 244 μM, respectively. This indicates that the antibacterial effect of the AMPs designed by us is stronger than that of most studied AMPs. Among our AMPs, S-6 with added pheromone, enhanced the antibacterial effect against the Gram-negative bacteria *P. mendocina* and *V. parahaemolyticus*, which may be because of the fact that the peptide chain contains a pair of disulfide bonds, which can increase the stability of AMPs and improve bioavailability (Nehls et al., 2020). Among the AMPs, P-6, S-6, and L-6, with the addition of pheromones, showed enhanced bacteriostatic effects against Gram-negative bacteria *P. mendocina*, and P-6 and S-6 showed enhanced bacteriostatic effects against Gram-negative bacteria *V. parahaemolyticus*, which may be attributed to the existence of Lipopolysaccharides (LPS) on the cell membranes of Gram-negative bacteria.

**TABLE 4:** Minimum inhibitory concentration (MIC) values of several natural antibacterial peptides.

Bacterial strain	AMP (MIC μM)
*Pseudomonas aeruginosa*	Indolicidin (8) (Park et al., 2009)
*Staphylococcus epidermidis*	Indolicidin (8) (Park et al., 2009)
*Fusarium culmorum*	Rs-AFP2 (8.5) (Kumar et al., 2018)
*Pseudomonas aeruginosa*	Guavanin 2 (25) (Porto et al., 2018)
*Listeria ivanovii*	Guavanin 2 (50) (Porto et al., 2018)
*Pseudomonas aeruginosa*	Holothuroidin 2 (8064) (Schillaci et al., 2013)
*Staphylococcus aureus*	Holothuroidin 2 (8064) (Schillaci et al., 2013)

The potential modes of action of cationic AMPs include binding to or insertion in bacterial membranes, thereby causing the depolarization of normally polarized membranes, forming physical pores that disrupt the normal distribution of bilayer lipids, or disrupting key intracellular targets. Using the membrane potential indicator DISC_3_-5, we demonstrated that membranes of bacterial cells exposed to the four AMPs were depolarized. PI is a fluorescent dye that inserts into DNA but does not penetrate intact cell membranes. Additionally, when bacterial cells were incubated with PI, we observed an increase in the fluorescence ratio in the cells treated with the four AMPs, indicating membrane permeabilization (Mortimer et al., 2000) and plasma membrane disruption (Pilsczek et al., 2010). This was also confirmed by the induction of AMPs into the cell membrane and subsequent rupture of *V. anguillarum*, *M. luteus*, and *L. monocytogenes* (Figures 4–6 and Supplementary Figure 14). High intracellular ROS levels can cause cell necrosis or apoptosis. The results of ROS level detection showed that AMPs can significantly increase the level of ROS in bacterial cells, which may induce cell necrosis or apoptosis (Figure 9).

A key point in the development of membrane-soluble antimicrobial therapies is that they must not damage the membranes of mammalian cells. Our results indicated that P-6 exhibited relatively high toxicity to mammalian cell membranes, possibly because of an excessive positive charge. The positive charge of AMPs was positively correlated with the toxicity to erythrocytes. Peptides with a charge higher than +9 reportedly damage red blood cells (Lima et al., 2021). However, compared with the antimicrobial activity, the four AMPs showed little cytotoxic activity against mouse macrophage RAW264.7. This indicates that the designed AMPs have membrane selectivity toward bacterial cells.

The gut microbiota have many important functions in body, including supporting resistance to pathogens, affecting the immune system (Chung et al., 2012; Arpaia et al., 2013), and affecting the behavioral and neurological functions of the host (Buffington et al., 2016; Zheng et al., 2019). The perturbation of the gut microbiota population associated with several human diseases that include inflammatory bowel diseases (IBD) (Takahashi et al., 2016; Nishino et al., 2018), obesity and diabetes (Karlsson et al., 2013).

Each class of antibiotics has different properties and excretion systems, resulting in different patterns of alteration to the microbiome. Ciprofloxacin reduced Firmicutes and Actinobacteria (especially Bifidobacterium), and increased *Bacteroides* (Stewardson et al., 2015). A Finnish report demonstrated that macrolide consumption in children led to an alteration in gut microbiota that decreased Actinobacteria and increased Bacteroides abundance. Research on the effects of AMPs on gut microbiota is of great significance for reducing the generation of drug-resistant bacteria, protecting gut microbiota, and reducing gastrointestinal side effects, and has important clinical and application value.

This study used MiSeq 16S rRNA high-throughput sequencing and bioinformatics methods, and analyses the influence of antibacterial peptides and antibiotic exposure after the *S. schlegelii* intestinal structure and change, the influence of alpha diversity analysis found that as well, the effect of exposure after the *S. schlegelii*, intestinal flora, the marked increase in abundance and diversity of 1–6, L-6 showed no significant change. At the family level, the relative abundance of *Lactobacillaceae* in groups 1–6 and L-6 increased. Wang et al. (2015) verified that Lactobacillus fermentum NS9 had a certain recovery effect on the in-field and out-field changes caused by antibiotics. This suggests that 1–6 and L-6 play a protective role in intestinal flora.

In summary, this study highlights the design of AMPs with higher charges and proportions of hydrophobic residues by replacing amino acids at selected locations in mBjAMP1 with tryptophan and lysine and adding pheromones at the N-terminal. Our experimental results further demonstrated the ideal antibacterial activity, and further verified the effects of 1–6 and L-6 on the stability of fish intestinal flora. Our results indicate that AMPs have great potential as a novel peptide antimicrobial agent against aquatic animal pathogens. Our study provides a solution for the development of new aquatic antibiotic substitutes.

## Data Availability Statement

The original contributions presented in the study are publicly available. This data can be found here: https://www.ncbi.nlm.nih.gov/bioproject/PRJNA762512.

## Ethics Statement

All experimental procedures complied with the National Institute of Health Guide for the Care and the Use of Laboratory Animals and were approved by the Animal Care Committee of Ocean University of China. The exposure experiments were performed in semistatic conditions according to OECD Guidelines 210.35 Ice bath anesthesia was used for all experiments.

## Author Contributions

RT, PC, and SR designed the study and revised the manuscript. RT and MW performed the experiments. RT and PC analyzed and evaluated the data. LQ, HX, and JW helps raise the *Sebastes schlegelii*. All authors read and approved the final manuscript.

## Conflict of Interest

The authors declare that the research was conducted in the absence of any commercial or financial relationships that could be construed as a potential conflict of interest.

## Publisher’s Note

All claims expressed in this article are solely those of the authors and do not necessarily represent those of their affiliated organizations, or those of the publisher, the editors and the reviewers. Any product that may be evaluated in this article, or claim that may be made by its manufacturer, is not guaranteed or endorsed by the publisher.

## Footnotes

1
https://web.expasy.org/protparam/

2
https://zhanglab.ccmb.med.umich.edu/

3
http://lbqp.unb.br/NetWheels/
