# Supplementary material for: Improving the Activity of Antimicrobial Peptides Against Aquatic Pathogen Bacteria by Amino Acid Substitutions and Changing the Ratio of Hydrophobic Residues
Source: Front Microbiol. 2021 Oct 18;12:773076. doi: 10.3389/fmicb.2021.773076 (PMC8558516; doi:10.3389/fmicb.2021.773076)
Supplement: Supplementary file 1 [file Data_Sheet_1.docx]

Improving the Activity of Antimicrobial Peptides against Aquatic pathogen bacteria by Amino Acid Substitutions and Changing the Ratio of Hydrophobic Residues

**
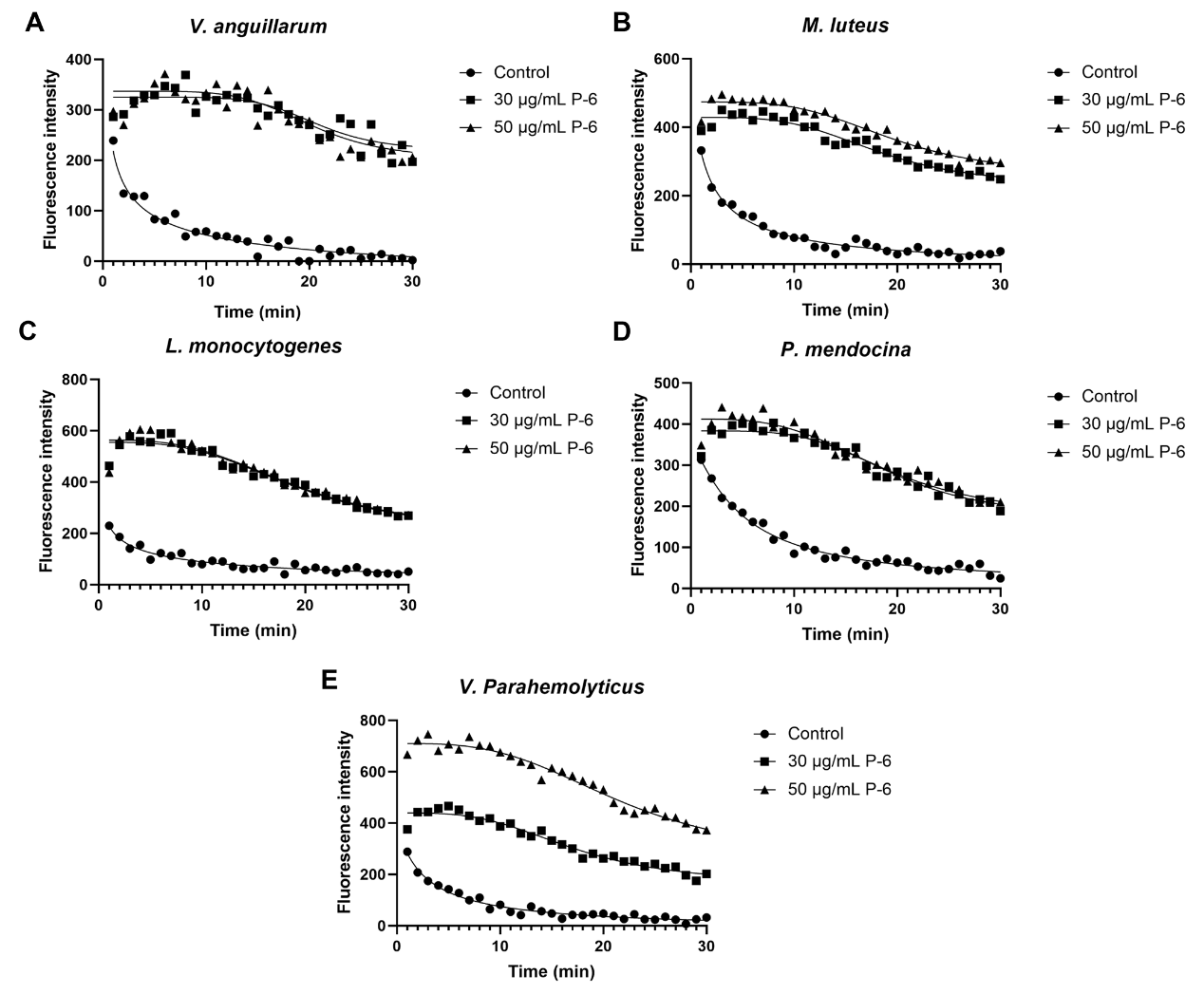
**

**Supplementary** **Figure 1. Bacterial membrane depolarization.** Depolarization of the bacterial membrane after incubation with antimicrobial peptides P-6 was detected using DiSC3-5 (excitation, 622 nm; emission, 670 nm).


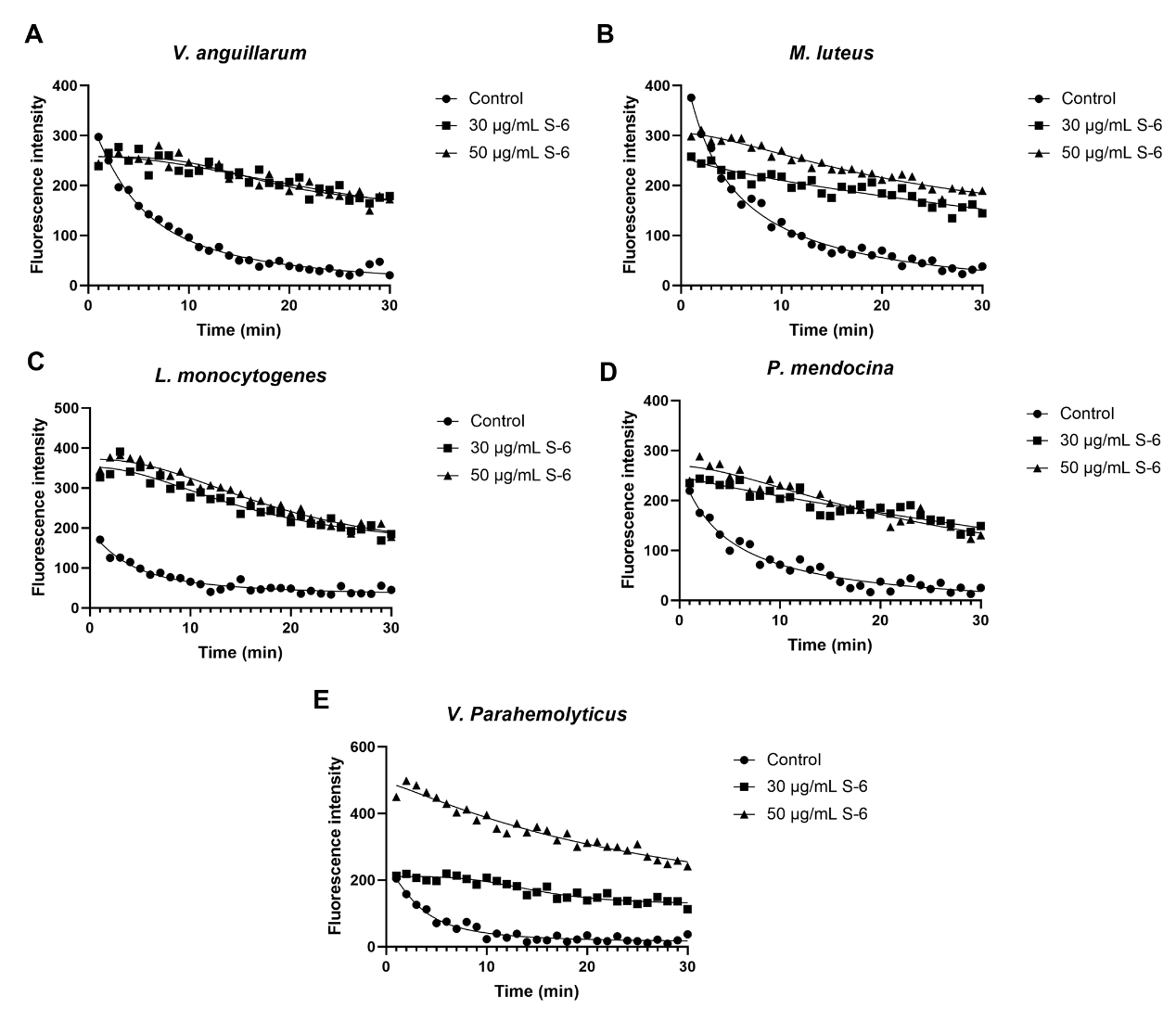


**Supplementary Figure 2. Bacterial membrane depolarization.** Depolarization of the bacterial membrane after incubation with antimicrobial peptides S-6 was detected using DiSC3-5 (excitation, 622 nm; emission, 670 nm).


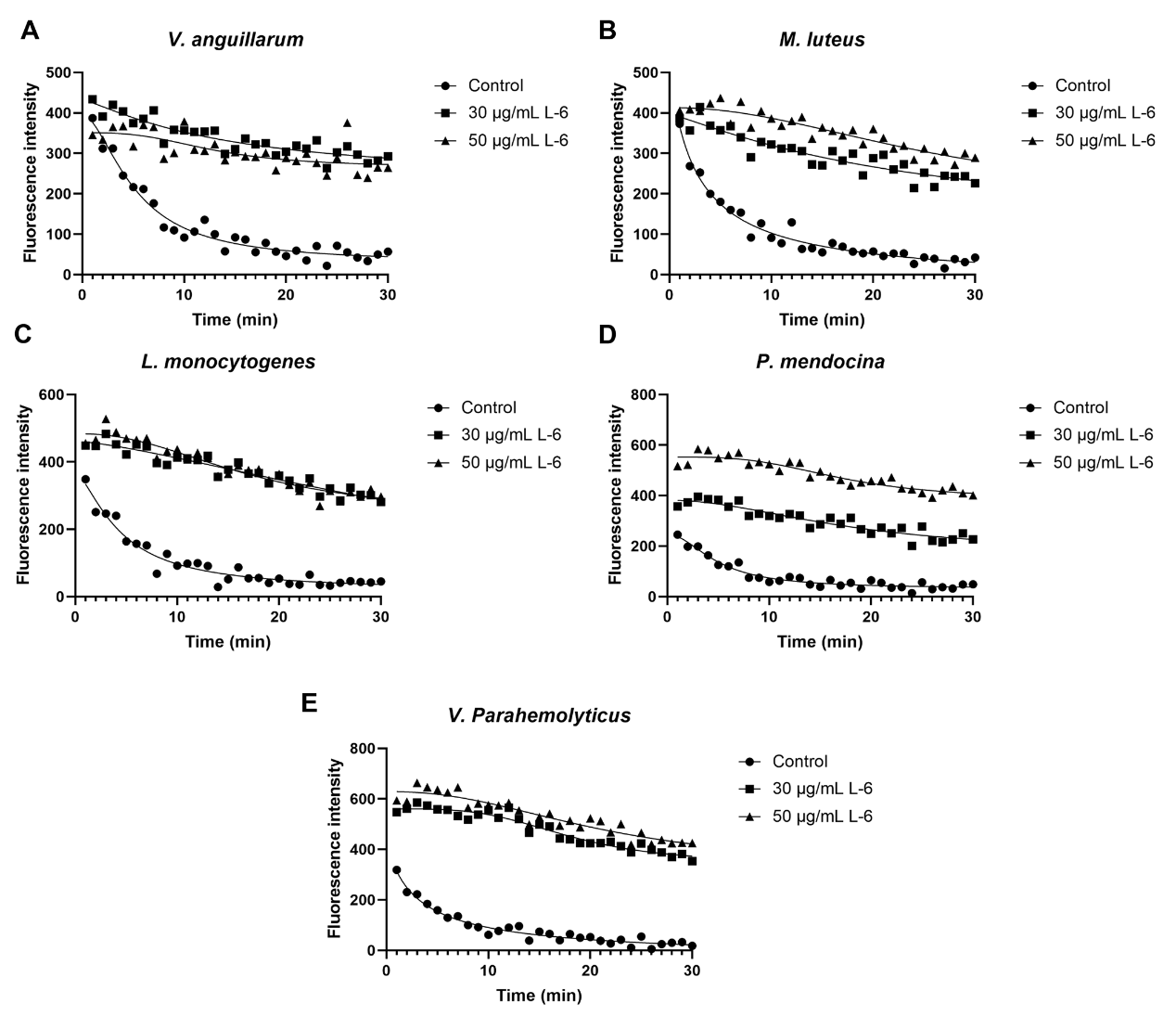


**Supplementary Figure 3. Bacterial membrane depolarization.** Depolarization of the bacterial membrane after incubation with antimicrobial peptides L-6 was detected using DiSC3-5 (excitation, 622 nm; emission, 670 nm).


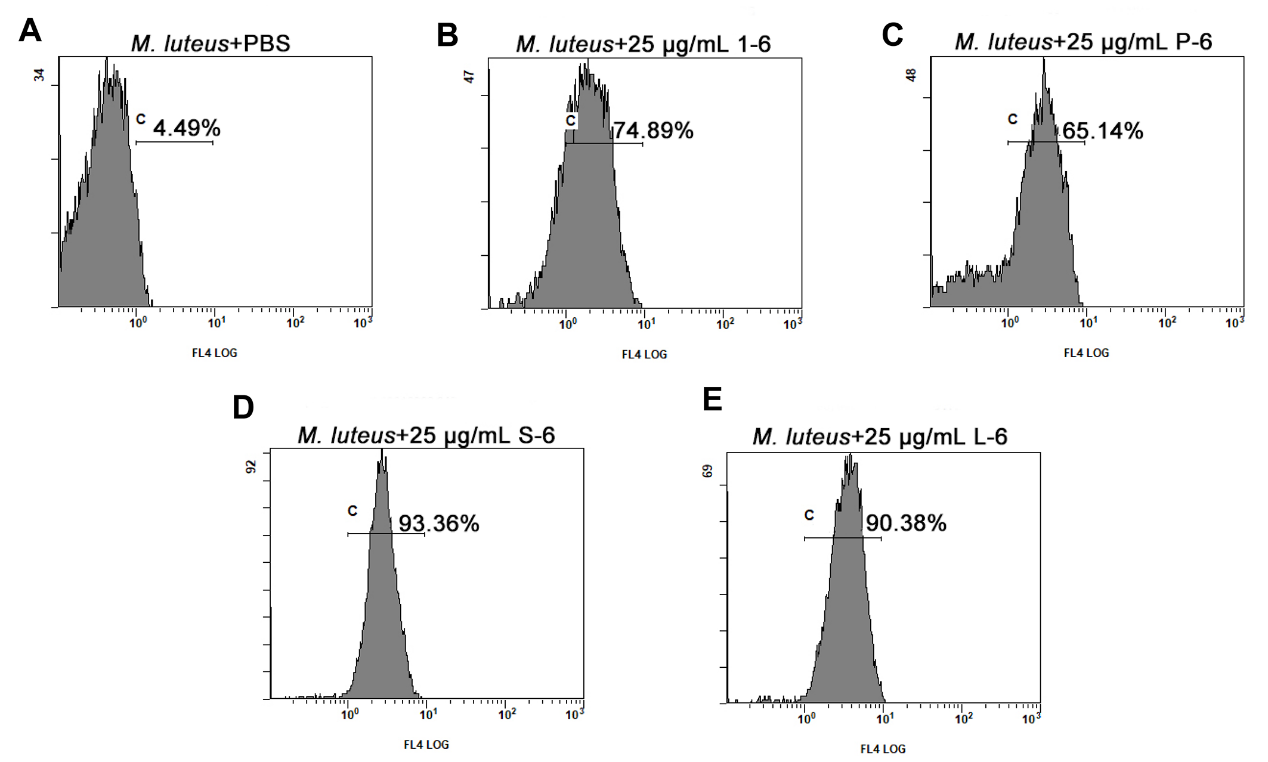


**Supplementary Figure 4.** **Membrane permeation of *M. luteus.*** The effects of 25 μg/ml of 1-6, P-6, S-6 and L-6 on the membrane integrity of the *M. luteus* cells were analyzed by flow cytometry. The proportion of integrated cells was shown near c.


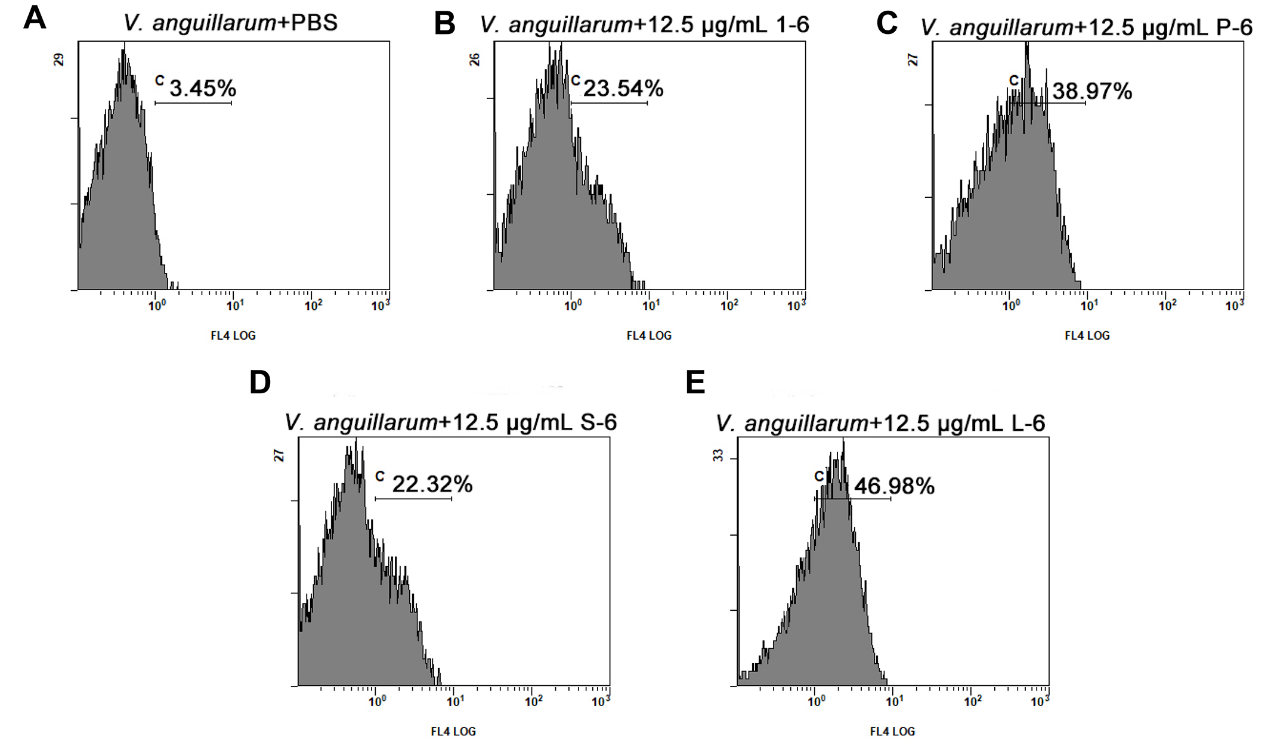


**Supplementary Figure 5.** **Membrane permeation of** ***V. anguillarum.*** The effects of 12.5 μg/ml of 1-6, P-6, S-6 and L-6 on the membrane integrity of the *V. anguillarum* cells were analyzed by flow cytometry. The proportion of integrated cells was shown near c.


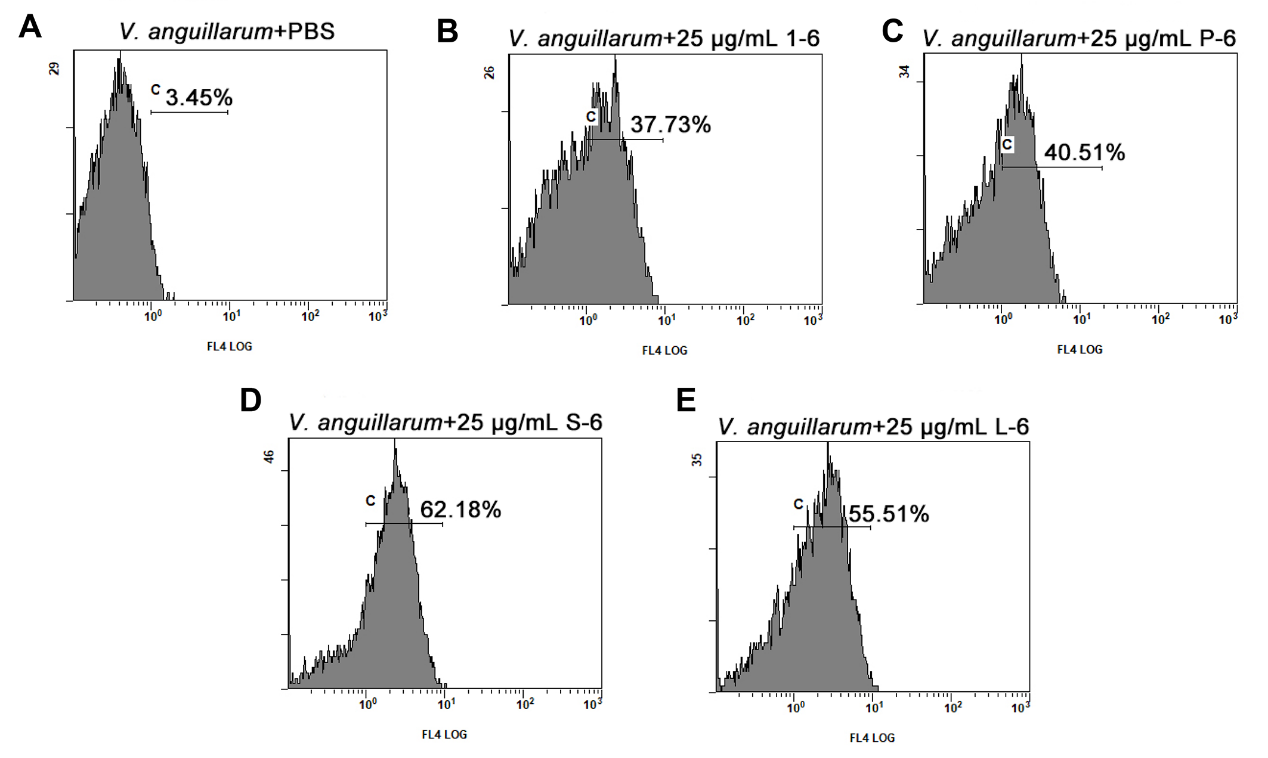


**Supplementary Figure 6.** **Membrane permeation of *V. anguillarum.*** The effects of 25 μg/ml of 1-6, P-6, S-6 and L-6 on the membrane integrity of the *V. anguillarum* cells were analyzed by flow cytometry. The proportion of integrated cells was shown near c.


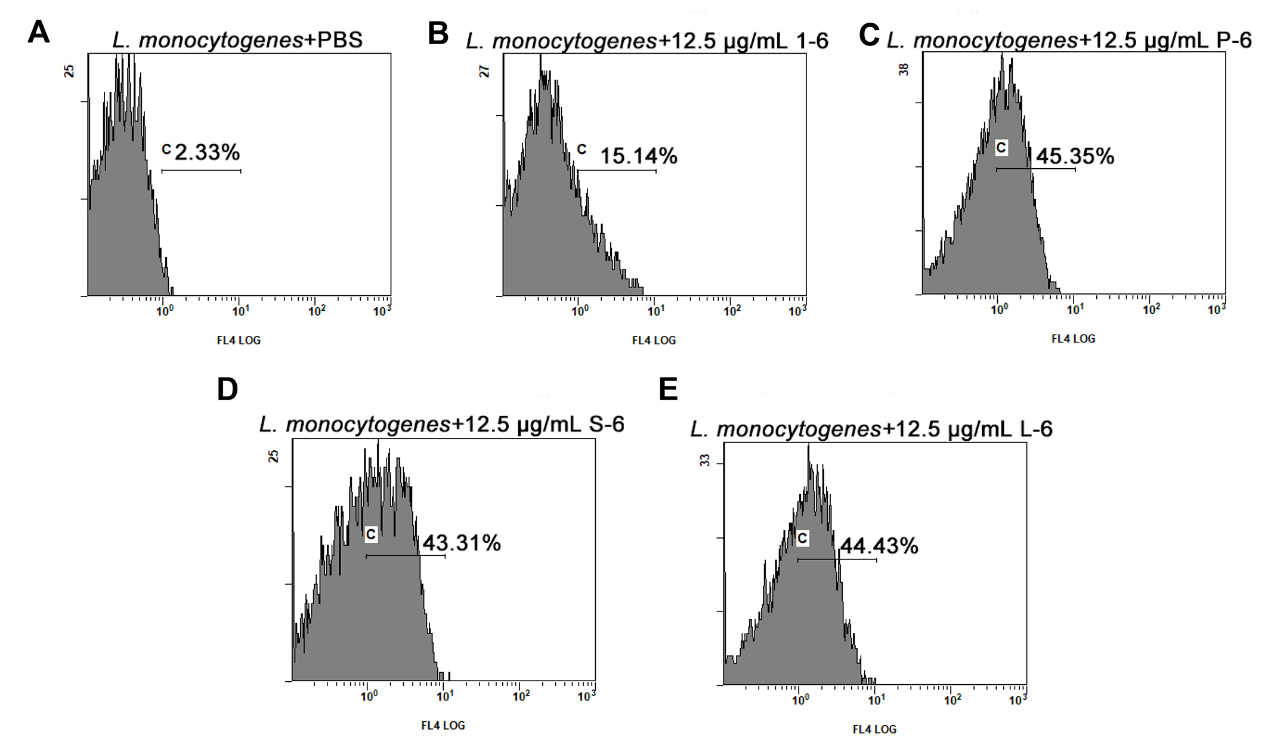


**Supplementary Figure 7. Membrane permeation of *L. monocytogenes.*** The effects of 12.5 μg/ml of 1-6, P-6, S-6 and L-6 on the membrane integrity of the *L. monocytogenes* cells were analyzed by flow cytometry. The proportion of integrated cells was shown near c.


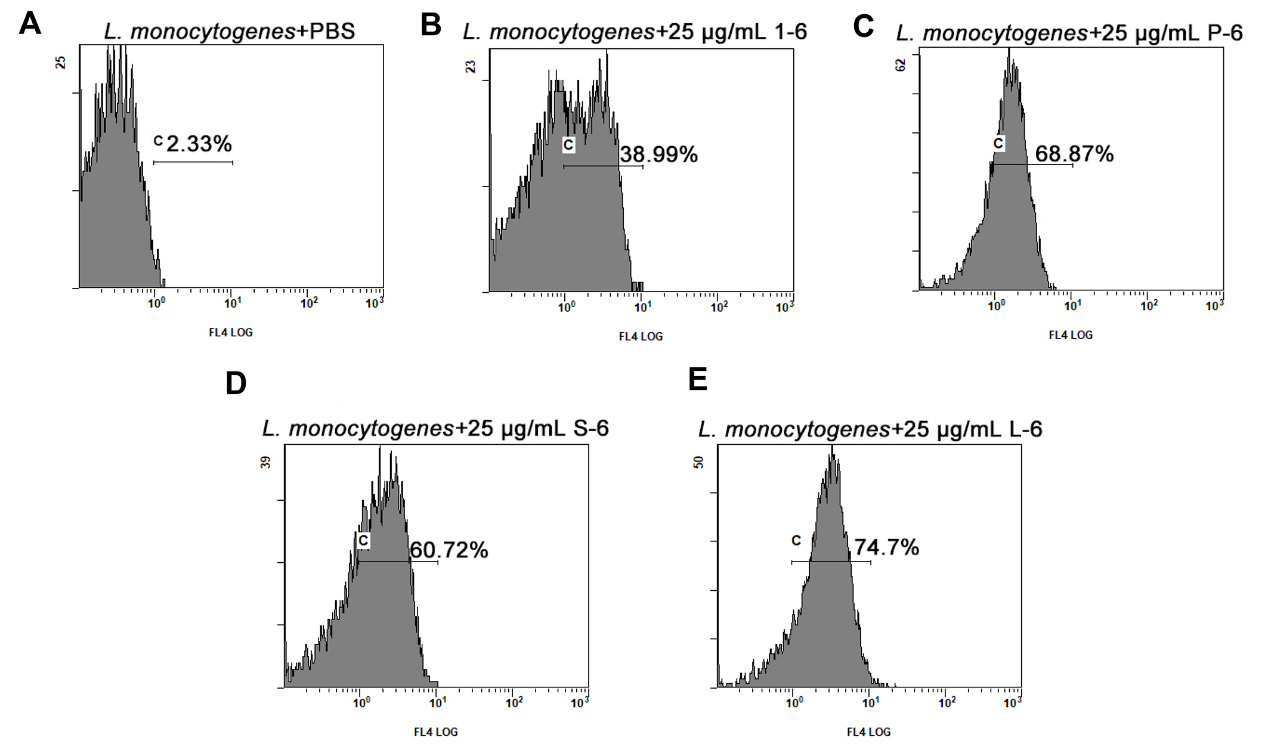


**Supplementary Figure 8. Membrane permeation of *L. monocytogenes.*** The effects of 25 μg/ml of 1-6, P-6, S-6 and L-6 on the membrane integrity of the *L. monocytogenes* cells were analyzed by flow cytometry. The proportion of integrated cells was shown near c.


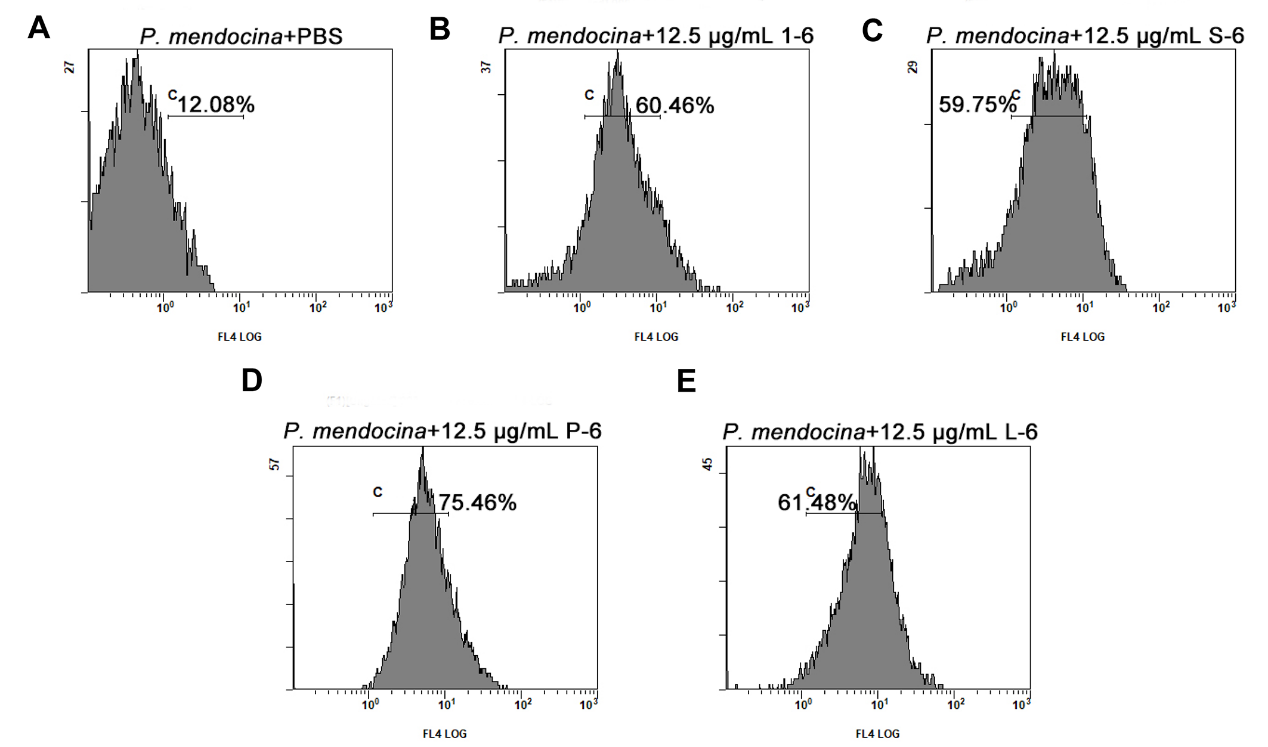


**Supplementary Figure 9. Membrane permeation of** ***P. mendocina.*** The effects of 12.5 μg/ml of 1-6, P-6, S-6 and L-6 on the membrane integrity of the *P. mendocina* cells were analyzed by flow cytometry. The proportion of integrated cells was shown near c.


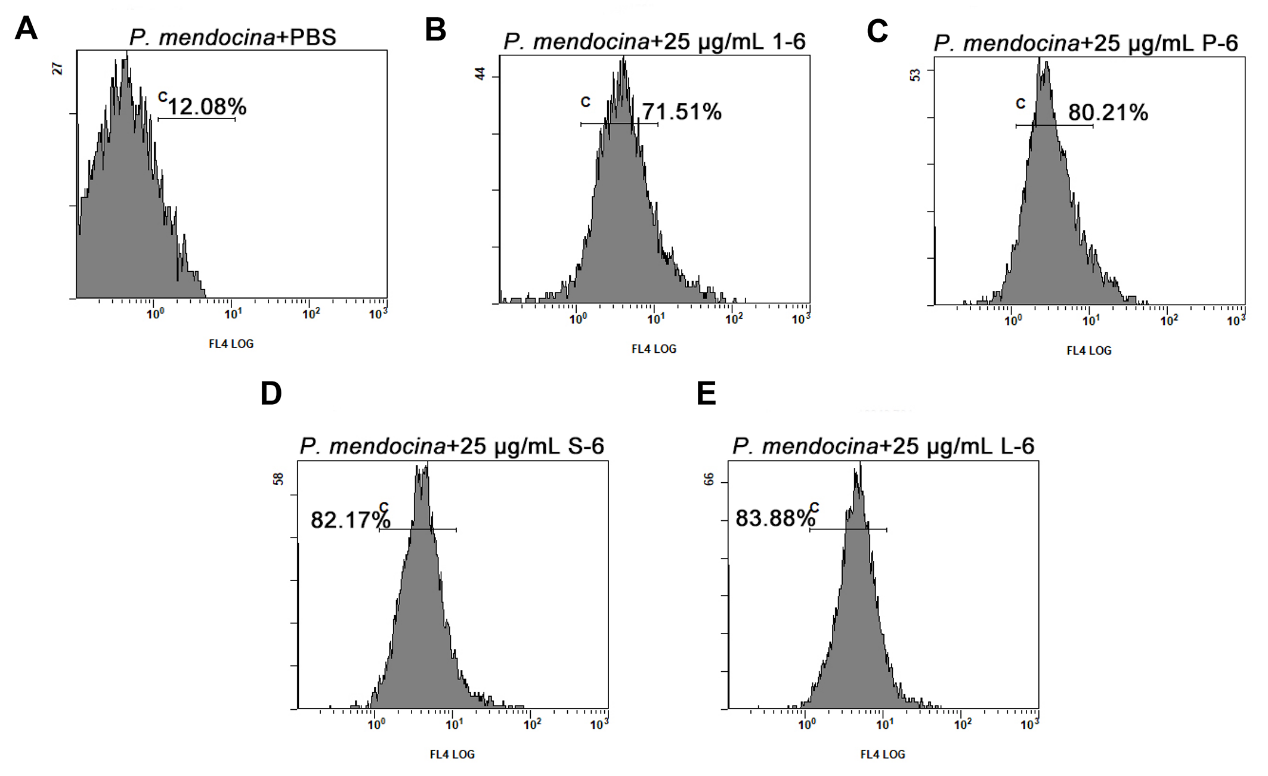


**Supplementary Figure 10. Membrane permeation of *P. mendocina.*** The effects of 25 μg/ml of 1-6, P-6, S-6 and L-6 on the membrane integrity of the *P. mendocina* cells were analyzed by flow cytometry. The proportion of integrated cells was shown near c.


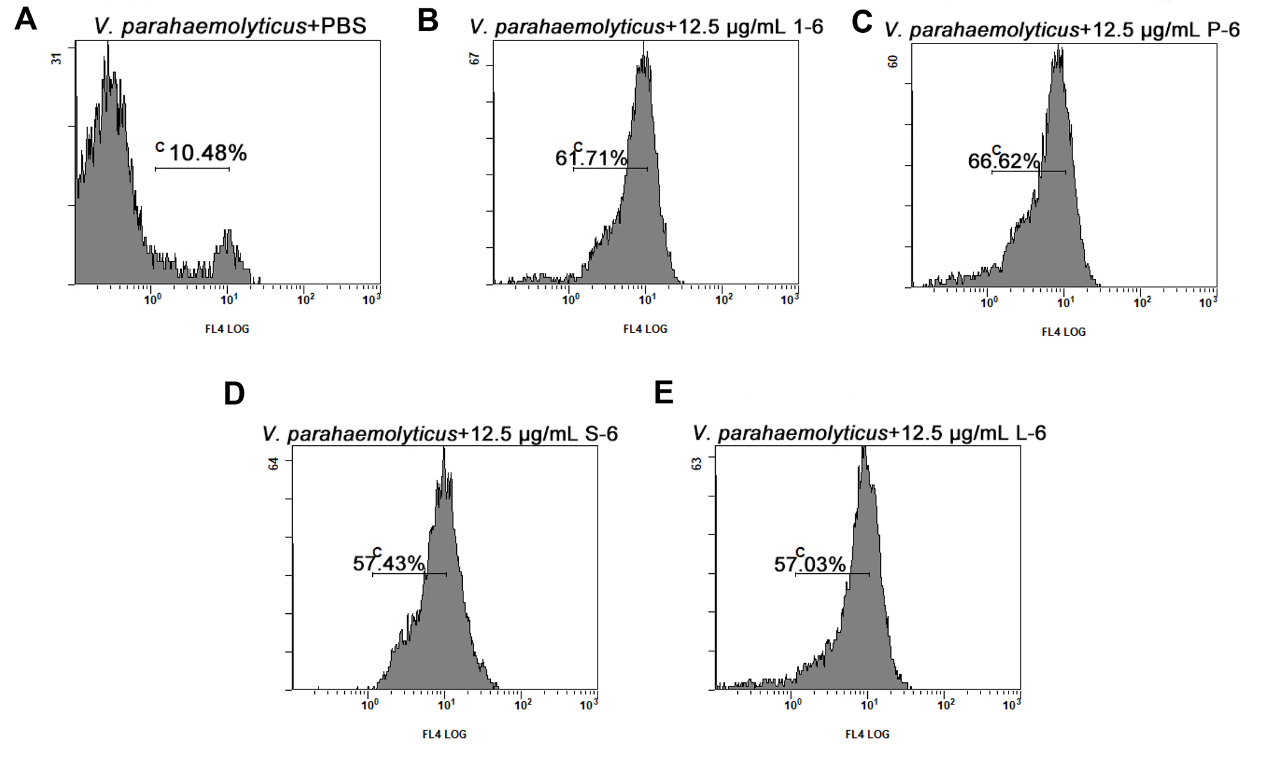


**Supplementary Figure 11. Membrane permeation of** ***V. parahaemolyticus.*** The effects of 12.5 μg/ml of 1-6, P-6, S-6 and L-6 on the membrane integrity of the *V. parahaemolyticus* cells were analyzed by flow cytometry. The proportion of integrated cells was shown near c.


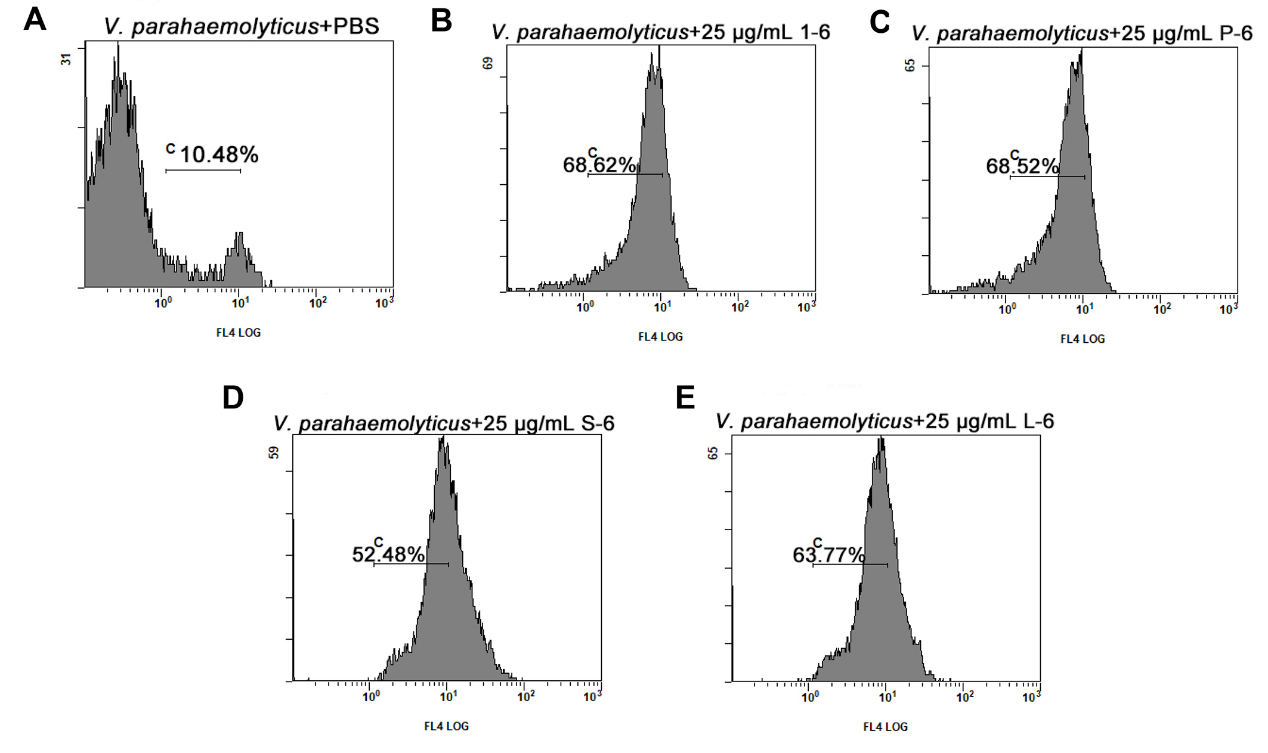


**Supplementary Figure 12. Membrane permeation of *V. parahaemolyticus.*** The effects of 25 μg/ml of 1-6, P-6, S-6 and L-6 on the membrane integrity of the *V. parahaemolyticus* cells were analyzed by flow cytometry. The proportion of integrated cells was shown near c.


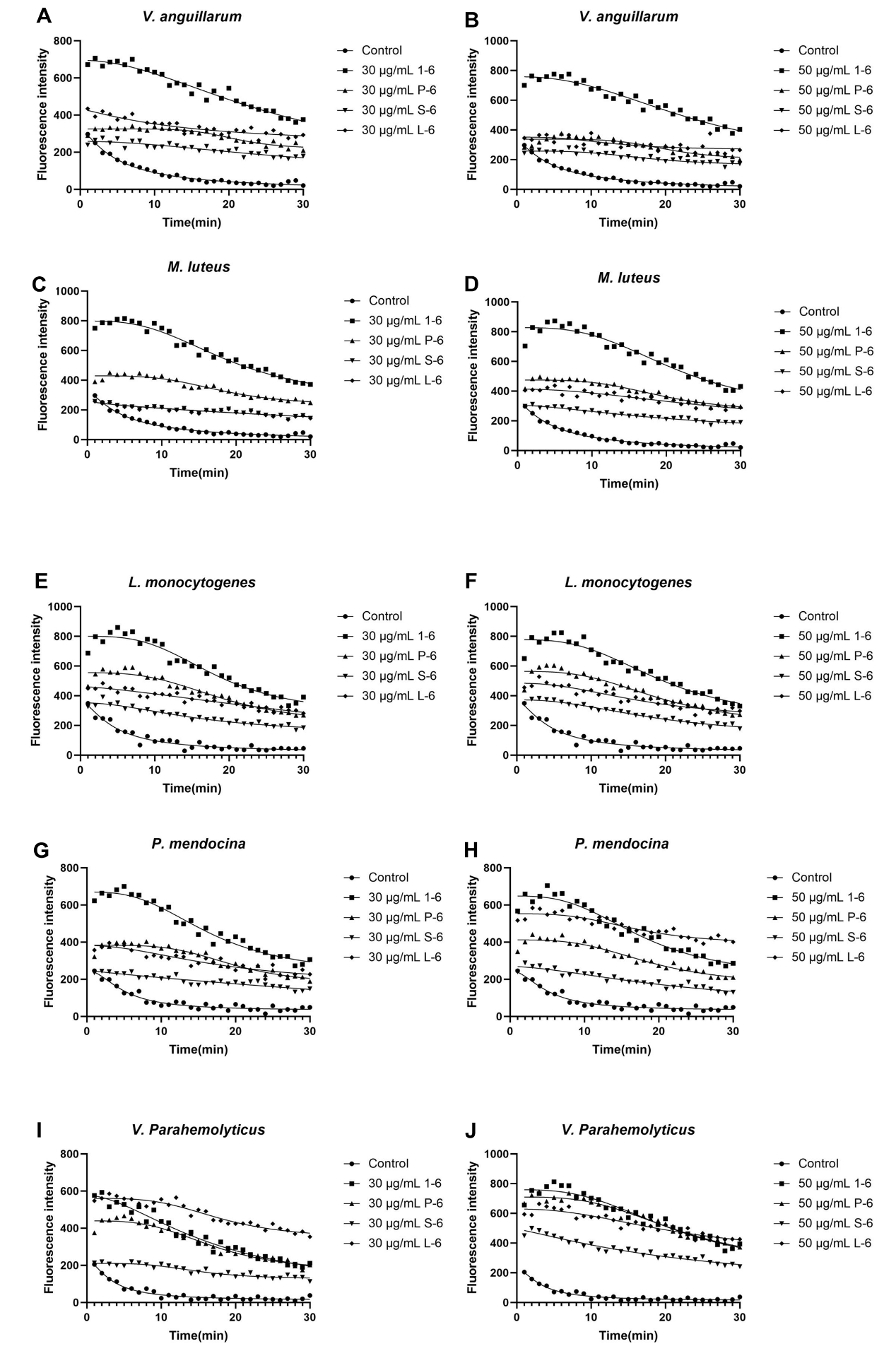


**Supplementary Figure 13. Bacterial membrane depolarization.** Depolarization of the bacterial membrane after incubation with antimicrobial peptides was detected using DiSC_3_-5 (excitation, 622 nm; emission, 670 nm).


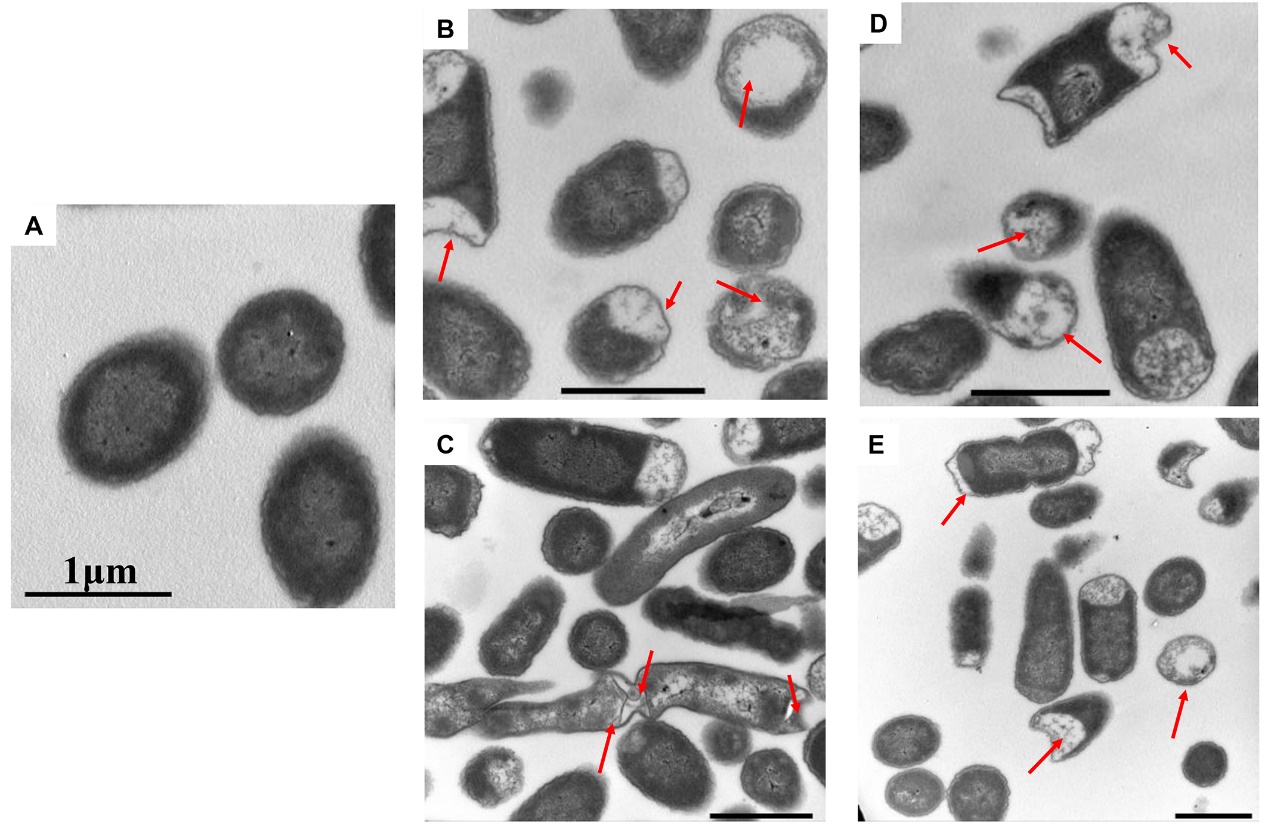


**Supplementary Figure 14.** **Ultra-thin section of *M. luteus* for electron microscopic observation.** *M. luteus* incubated with PBS (**A**) or with 1-6(**B**), P-6(**C**), S-6(**D**), and L-6 (**E**) at 28 °C for 30 min.
